# Neuropsychological functioning after COVID-19: Minor differences between individuals with and without persistent complaints after SARS-CoV-2 infection

**DOI:** 10.1080/13854046.2024.2379508

**Published:** 2024-07-17

**Authors:** Anouk Verveen, Sander C.J Verfaillie, Denise Visser, Dook W. Koch, Esmée Verwijk, Gert J. Geurtsen, Jeroen Roor, Brent Appelman, Ronald Boellaard, Caroline M. van Heugten, Janneke Horn, Hanneke E. Hulst, Menno D. de Jong, Tanja A. Kuut, Tessa van der Maaden, Yvonne M.G van Os, Maria Prins, Johanna M.A Visser-Meily, Michele van Vugt, Cees C. van den Wijngaard, Pythia T. Nieuwkerk, Bart van Berckel, Nelleke Tolboom, Hans Knoop

**Affiliations:** a Department of Medical Psychology, Amsterdam UMC location University of Amsterdam, Amsterdam, The Netherlands; b Amsterdam Public Health, Amsterdam, The Netherlands; c Radiology & Nuclear Medicine, Amsterdam UMC location Vrije Universiteit Amsterdam, Amsterdam, The Netherlands; d Amsterdam Neuroscience, Amsterdam, The Netherlands; e GGz inGeest Specialized Mental Health Care, Amsterdam, The Netherlands; f Department of Radiology and Nuclear Medicine, Division of Imaging and Oncology, University Medical Center Utrecht, Utrecht, The Netherlands; g Psychology department, Brain and Cognition, University of Amsterdam, Amsterdam, The Netherlands; h Department of Medical Psychology, VieCuri Medical Center, Venlo, The Netherlands; i School for Mental Health and Neuroscience, Maastricht University, Maastricht, The Netherlands; j Center for Experimental and Molecular Medicine, Amsterdam UMC location University of Amsterdam, Amsterdam, The Netherlands; k Department of Neuropsychology and Psychopharmacology, and Limburg Brain Injury Center, Faculty of Psychology, Neuroscience Maastricht University, Maastricht, The Netherlands; l Intensive Care, Amsterdam UMC location University of Amsterdam, Amsterdam, The Netherlands; m Department of Medical, Health and Neuropsychology, Leiden University, Leiden, The Netherlands; n Infectious Diseases, Amsterdam UMC location University of Amsterdam, Amsterdam, The Netherlands; o Medical Microbiology & Infection Prevention, Amsterdam UMC location University of Amsterdam, Amsterdam, The Netherlands; p Center for Infectious Disease Control, National Institute for Public Health and the Environment (RIVM), Bilthoven, The Netherlands; q Occupational Health Office, Department of Human Resources, University Medical Center Utrecht, Utrecht, The Netherlands; r Infectious Diseases, Amsterdam Institute for Infection and Immunity, Amsterdam, The Netherlands; s Department of Infectious Diseases, Public Health Service of Amsterdam, Amsterdam, The Netherlands; t Department of Rehabilitation, Physical Therapy Science and Sports, University Medical Centre Utrecht, Utrecht, The Netherlands; u Internal Medicine, Amsterdam UMC location University of Amsterdam, Amsterdam, The Netherlands

**Keywords:** Post-COVID PASC severe fatigue brain fog neuropsychology cognitive functioning

## Abstract

**Objective:** It is unclear how self-reported severe fatigue and difficulty concentrating after SARS-CoV-2 infection relate to objective neuropsychological functioning. The study aimed to compare neuropsychological functioning between individuals with and without these persistent subjective complaints. **Method**: Individuals with and without persistent severe fatigue (Checklist Individual Strength (CIS) fatigue ≥ 35) and difficulty concentrating (CIS concentration ≥ 18) at least 3 months after SARS-CoV-2 infection were included. Neuropsychological assessment was performed on overall cognitive functioning, attention, processing speed, executive functioning, memory, visuo-construction, and language (18 tests). T-scores −1.5 SD below population normative data (*T* ≤ 35) were classified as “impaired”. **Results:** 230 participants were included in the study, of whom 22 were excluded from the analysis due to invalid performance. Of the participants included in the analysis, 111 reported persistent complaints of severe fatigue and difficulty concentrating and 97 did not. Median age was 54 years, 59% (*n* = 126) were female, and participants were assessed a median of 23 months after first infection (IQR: 16–28). With bivariate logistic regression, individuals with persistent complaints had an increased likelihood of slower information processing speed performance on the Stroop word reading (OR = 2.45, 95%CI = 1.02–5.84) compared to those without persistent complaints. Demographic or clinical covariates (e.g. hospitalization) did not influence this association. With linear regression techniques, persistent complaints were associated with lower t-scores on the D2 CP, TMT B, and TMT B|A. There were no differences in performance on the other neuropsychological tests. **Conclusions:** Individuals with subjective severe fatigue and difficulty concentrating after COVID-19 do not typically demonstrate cognitive impairment on extensive neuropsychological testing.

## Introduction

Severe fatigue and difficulty concentrating are among the most common persistent complaints after infection with SARS-CoV-2, with prevalence rates of 15 to 45% up to 12 months after infection and are associated with lowered health-related quality of life and functional limitations (Ceban et al., 2022). Patients with post-acute sequelae of COVID-19 (PASC) may report various cognitive complaints, such as brain fog or concentration and memory problems (Crivelli et al., 2022). So far, most studies investigating cognitive functioning have primarily investigated overall cognition, were limited to individuals with severe initial COVID-19 and/or were conducted at a relatively short time since illness onset of COVID-19 (<1 year) (Zhao et al., 2023; Schild et al., 2023; Sobrino-Relaño et al., 2023). Studies using objective cognitive data are necessary to identify whether there is specific cognitive dysfunction associated with PASC (Becker et al., 2023).

Previous neuropsychological studies demonstrated cognitive deficits in attention, executive functioning, processing speed, verbal fluency, and memory up to 12 months after SARS-CoV-2 infection in a subset of patients (García-Sánchez et al., 2022; Delgado-Alonso et al., 2022; Bungenberg et al., 2022), although these studies did not control for performance below best of capabilities (i.e. invalid performance), which is crucial to place confidence in the validity of cognitive test results (Conference Participants, 2021; Whiteside et al., 2024).

While some studies have focused on individuals with PASC (Morawa et al., 2023), most have not included a control group of individuals with previous SARS-CoV-2 infection but without PASC. One study linked post-COVID fatigue to decreased overall cognitive performance compared to controls (Mancilla-Corona et al., 2023). Severity of COVID-19 in the acute phase might be associated with subjective cognitive symptoms (Ceban et al., 2022) and possibly objective cognitive impairment, but to date studies have generated inconsistent results with respect to this relation (García-Sánchez et al., 2022; Bungenberg et al., 2022). Neuropsychological test performance may also be influenced by sociodemographic (e.g. age, sex), clinical (e.g. comorbidities, hospitalization in the acute phase of infection, depressive symptoms) and predisposing factors (e.g. apolipoprotein ɛ4 (ApoE-ɛ4) genotype) (MacAulay et al., 2020).

Taken together, it is currently unclear whether there are specific cognitive impairments as measured with extensive neuropsychological testing in individuals with persistent self-reported cognitive complaints, particularly among individuals who had initial mild COVID-19. This study aimed to investigate whether individuals with persistent (more than 3 months after infection) subjective severe fatigue and difficulty concentrating have more impaired cognitive performance on neuropsychological testing than individuals who do not report persistent complaints after COVID-19, while considering aforementioned relevant confounding factors.

## Materials and methods

### Study design

The VeCosCO study is a cross-sectional study in participants with a confirmed previous SARS-CoV-2 infection (Verveen et al., 2023). Briefly, individuals with and without persistent fatigue and difficulty concentrating following COVID-19 were included from six existing prospective COVID-19 cohorts. All cohorts included participants infected with SARS-CoV-2 between March 2020 and December 2021, except for one cohort for which inclusion is ongoing.

For inclusion in the VeCosCO study, participants’ most recent diagnosis of COVID-19 had to be at least 3 months from the inclusion date. To determine the presence of severe fatigue and self-reported difficulty concentrating, we used the Checklist Individual Strength (CIS) (Worm-Smeitink et al., 2017). The fatigue subscale has a validated cutoff of ≥35 (Worm-Smeitink et al., 2017), and for the concentration subscale, a cutoff threshold of ≥18 defines the presence of notable concentration problems. This cutoff is based on data from Worm-Smeitink et al and corresponds with 85% of healthy subjects scoring below this threshold (Verveen et al., 2023). According to these cutoff scores, participants were considered having “persistent complaints” (CIS fatigue ≥35 and CIS concentration ≥18) or “without persistent complaints” (CIS fatigue <35 and CIS concentration <18). Participants who scored above only one of the cutoffs were not eligible for inclusion. Additional exclusion criteria were (1) a current psychiatric or somatic condition that could explain the presence of complaints; (2) having severe fatigue or cognitive complaints prior to the first SARS-CoV-2 infection based on self-reported clinical history; (3) insufficient Dutch language proficiency. Moreover, individuals were not included in analyses if there was evidence for invalid neuropsychological test performance, using the Test of Memory Malingering (TOMM) with a pre-established cutoff (Trial 1 < 42) (Martin et al., 2020). In a sensitivity analysis we explored the impact of using more or less stringent cutoffs for the TOMM trial 1 (T1 < 40, T1 < 43 and T1 < 44) and the use of both TOMM trials (T1 < 45 & T2 < 45).

During a single study visit, participants underwent an extensive neuropsychological assessment, several physical tests and blood sampling. Additionally, we asked participants to complete an online battery of questionnaires (Verveen et al., 2023). The medical ethical review board of the Amsterdam University Medical Centers approved the VeCosCO study (NL79575.018.21). All participants provided written informed consent.

#### Neuropsychological assessment

The Montreal Cognitive Assessment (MoCA) was used to assess overall cognitive functioning (Nasreddine et al., 2005). Subsequently, neuropsychological assessment was performed for the following cognitive domains (Harvey, 2019): *Attention*, measured with the D2 test (Bates & Lemay, 2004) and Digit-span forward (Cullum, 1998). The D2 test was used to measure sustained attention and concentration accuracy with three parameters: total number of items processed (TN), percentage of errors (E%) and concentration performance (CP). *Processing speed* was measured with the Stroop word reading, Stroop colour naming (Stroop, 1992) and Trail Making Test (TMT) part A (Llinàs-Reglà et al., 2017). *Executive functioning* was measured with the Controlled Oral Word Association Test (COWAT) for verbal fluency (Schmand et al., 2008), the Digit-span backward for working memory skills (Cullum, 1998), Stroop Colour-Word Test for cognitive interference (SCWT) (Stroop, 1992), and TMT-B and TMT B|A for set switching and mental flexibility (Llinàs-Reglà et al., 2017). Both *verbal and visual memory* were assessed with the Dutch translation of the Rey Auditory Verbal Learning test (RAVL immediate and delayed recall) (Saan & Deelman, 1986) and the recall condition of the Rey Complex Figure Test (CFT) (Rey, 1941). *Visuo-construction* was assessed with the copy condition of the CFT (Rey, 1941). *Language* was assessed through the Animal Fluency test (Ardila et al., 2006).

We extracted T-scores based on normative data adjusted for age, sex, and education level for all neuropsychological tests, except for the MoCA and CFT. Dutch norms are developed by the Netherlands Institute for Psychologists and were last updated in 2012 (Schmand et al., 2012). In this study, scores −1.5 SD from population normative data (*T* ≤ 35) fall in a range that indicates cognitive impairment.

#### Potential sociodemographic and clinical confounders

Educational level was categorized based on the Dutch Verhage scale as low (up to low-level secondary education), middle (average-level secondary education), and high educational level (high-level secondary education or university degree) (Verhage, 1965).

Total number of the following self-reported comorbidities (before the first COVID-19 diagnosis) was calculated: cardiovascular disease (including hypertension), chronic pulmonary disease (including asthma), diabetes, neurological disease, haematological disease, rheumatic disease, thyroid disorders, renal disease, liver disease, cancer, and psychiatric illness.

Participants were considered of non-Dutch ethnic origin if they were born outside the Netherlands and at least one parent was born outside the Netherlands; or they were born in the Netherlands but both parents were born outside the Netherlands (Stronks et al., 2009).

Participants were considered completely vaccinated after receiving at least two doses of vaccine for BNT162b2 Pfizer, mRNA-1273 Moderna, or Oxford-AstraZeneca, or one dose for Janssen before the date of first SARS-CoV-2 infection.

Genomic DNA was extracted from the blood sample and ApoE genotyping was performed. Participants were dichotomized into two groups: ApoE-ɛ4 carriers (at least one copy of the ApoE ɛ4 allele) or non ɛ4-carriers (MacAulay et al., 2020).

Depressive symptoms were measured using the Hospital Anxiety and Depression Scale (HADS) (Spinhoven et al., 1997). The sum of 7 items (4-point scale) is the total score with cutoff threshold ≥8 on the depression subscale indicating the possible presence of a depressive disorder.

The Cognitive Failure Questionnaire (CFQ) was used to measure subjective cognitive functioning. The CFQ consists of 25 questions (range 1 (very often) to 5 (never)) about the frequency of everyday cognitive failures (Ponds et al., 2006). The total score is computed by reverse scoring the items. A cutoff threshold of ≥44 defines a heightened frequency of cognitive failures (Ponds et al., 2006).

Sleep problems are assessed using the Insomnia Severity Index (ISI) and Pittsburgh Sleep Quality Index (PSQI). For the ISI, the total score of 7 items (5-point scale) has a cutoff threshold ≥10 for insomnia (Morin et al., 2011). The PSQI consists of 19 items with different response options. A computed total score >5 indicates poor sleep quality (Buysse et al., 1989).

The Post-COVID-19 Functional Status Scale (PCFS) was used to evaluate the consequences of COVID-19 and their effect on functional status (Machado et al., 2021). It is composed of five scale grades ranging from 0 (No functional limitations) to grade 4 (Severe functional limitations).

### Statistical analysis

Data were entered into a secured electronic case report form (Castor EDC). Statistical analyses were performed using Stata (v.15.1, College Station, TX, USA). The required sample size was calculated with an α of 0.025 and β of 0.80, ­assuming a difference on the MoCA score of a medium sized magnitude (Cohen’s *d* = 0.5) between participants with and without persistent complaints (Verveen et al., 2023). Based on these assumptions, a sample size of *n* > 78 per group was required.

Sociodemographic and clinical factors are presented per group. Differences between patients with and without persistent symptoms were determined using Kruskal-Wallis and Pearson χ2 tests. We first calculated the number of neuropsychological tests on which patients scored below *T* = 36 and the median difference between the group with and without persistent complaints using Kruskal-Wallis test. We subsequently investigated whether persistent complaints (yes/no) were associated with an increased likelihood of scores indicating cognitive impairment (dichotomous, *T* ≤ 35) per neuropsychological test (except for the MoCA and CFT) using logistic regression analyses. We explored the effect of potential confounders on the association between the presence of persistent complaints and impaired neuropsychological test scores in series of logistic regression analysis. Impaired neuropsychological test score (yes/no) was the dependent variable, the presence of persistent complaints (yes/no) was kept in all analyses as independent variable to which we added one of the following potential confounders, i.e. variables that could both associated with the report of persistent symptoms as well as neuropsychological test performance, as additional independent variable: somatic comorbidities, psychiatric comorbidity, ApoE-ɛ4 genotype, migration background, vaccination status at time of infection, hospitalization for COVID-19, months since first infection and the presence of depressive symptoms (HADS ≥8). We explored the impact of potential confounders only if at least 20 participants had impaired neuropsychological test scores according to the rule of thumb that there should be at least 10 outcome events per predictor variable. As a sensitivity analysis, differences in T-scores of the neuropsychological tests were also analysed with the use of linear regression, also adjusted for the potential confounders. For the MoCA and CFT linear regression techniques were used. Two-sided *p* value <0.05 were considered statistically significant.

## Results

### Study population

230 participants were enrolled in the VeCosCO study, of whom 21 were excluded due to invalid test performance (9.1%), which would preclude a valid interpretation of neuropsychological test performance, and one participant due to insufficient Dutch language proficiency (0.4%). All 22 excluded participants reported severe fatigue and difficulty concentrating. Excluded participants did not differ significantly in age, sex, education level, number of comorbidities, and hospitalization or vaccination status from included participants with persistent complaints (Supplementary Table 1). Excluded participants were more often of non-Dutch origin than included participants with persistent complaints (32% vs. 13%; *p* = 0.021), more often had psychiatric comorbidity before COVID-19 (18% vs. 5%; *p* = 0.014) and more often reported clinically relevant levels of depressive symptoms (64% vs. 39%; *p* = 0.031).

Of the 208 participants included in the analysis, 118 (57%) were female and median age was 54 years [IQR 45–65]. About three quarters of participants (*n* = 152) had a high level of education. A third of participants (*n* = 62) had been hospitalized for COVID-19, of whom 24 (38.7%) were admitted to the Intensive Care Unit. Participants were assessed a median of 23 months after infection (IQR: 16–28). Participants with persistent fatigue and difficulty concentrating (*n* = 111) were significantly more often female (*p* < 0.001), younger (*p* < 0.001), of non-Dutch origin (*p* = 0.042), unfit for work (*p* < 0.001) and more often reported depressive symptoms (*p* < 0.001) than those without persistent complaints (*n* = 97) (Table 1). Significantly fewer individuals with persistent complaints were completely vaccinated against SARS-CoV-2 at the time of infection compared to individuals without persistent complaints (*p* = 0.005). Individuals with persistent complaints significantly more often scored above the cutoffs for cognitive failures (CFQ, 59%), poor sleep quality (PSQI, 70%), and insomnia (ISI, 54%) compared to those without persistent complaints (8%, 21% and 7%, respectively). Only two individuals (2%) without persistent complaints reported moderate to severe functional limitations on the PCFS, compared to 45 individuals (40%) with persistent complaints.

**Table 1.** Socio-demographic and clinical characteristics of VeCosCO study participants.

	Total group	Without persistent complaints^a^	With persistent complaints^a^	*p* Value
	*N* = 208	*N* = 97	*N* = 111	
Female sex	118 (57%)	39 (40%)	79 (71%)	<0.001
Age, years	54.0 (45.0–65.0)	61.0 (52.0–70.0)	50.0 (43.0–59.0)	<0.001
Highest level of education (Verhage)				0.24
Low	11 (5%)	7 (7%)	4 (4%)	
Middle	45 (22%)	17 (18%)	28 (25%)	
High	152 (73%)	73 (75%)	79 (71%)	
Current employment status				<0.001
Paid work or full-time student	129 (62%)	58 (60)	71 (64%)	
Unemployed or stay-at-home parent	10 (5%)	4 (4%)	6 (5%)	
Unfit for work	19 (9%)	0 (0%)	19 (17%)	
Retired	45 (22%)	34 (35%)	11 (10%)	
Missing	5 (2%)	1 (1%)	4 (4%)	
Non-Dutch origin^b^	19 (9%)	5 (5%)	14 (13%)	0.042
Missing	10 (5%)	1 (1%)	9 (8%)	
Number of comorbidities^c^				0.35
0	113 (54%)	54 (56%)	59 (53%)	
1	57 (27%)	24 (25%)	33 (30%)	
2	28 (13%)	16 (16%)	12 (11%)	
3 or more	8 (4%)	2 (2%)	6 (5%)	
Missing	2 (1%)	1 (1%)	1 (1%)	
Psychiatric comorbidity before COVID-19	10 (5%)	5 (5%)	5 (5%)	0.86
Missing	4 (2%)	1 (1%)	3 (3%)	
ApoE-ε4 genotype	63 (30%)	33 (34%)	30 (27%)	0.27
Missing	15 (7%)	7 (7%)	8 (7%)	
Months since first SARS-CoV-2 infection	23.0 (16.0–28.0)	24.0 (16.0–27.0)	23.0 (17.0–30.0)	0.18
Hospital admission for COVID-19	62 (30%)	30 (31%)	32 (29%)	0.74
ICU admission for COVID-19	24 (12%)	14 (14%)	10 (9%)	0.22
Vaccinated at time of infection	39 (19%)	26 (27%)	13 (12%)	0.005
Missing	10 (5%)	5 (5%)	5 (5%)	
CFQ total score	35.0 (22.0–49.0)	22.0 (16.0–33.0)	47.0 (35.5–57.0)	<0.001
Cognitive failures (CFQ ≥44)	73 (35%)	8 (8%)	65 (59%)	<0.001
PSQI total score	6.0 (3.0–9.0)	3.0 (2.0–5.0)	8.0 (6.0–11.0)	<0.001
Poor sleep quality (PSQI >5)	99 (48%)	21 (22%)	78 (70%)	<0.001
ISI total score	6.0 (3.0–11.0)	3.0 (1.0–6.0)	10.5 (7.0–14.0)	<0.001
Insomnia (ISI ≥10)	67 (32%)	7 (7%)	60 (54%)	<0.001
HADS total score	3.0 (1.0–6.0)	1.0 (0.0–2.0)	6.0 (3.0–9.0)	<0.001
Clinically relevant symptoms of depression (HADS ≥ 8)	46 (22%)	3 (3%)	43 (39%)	<0.001
Post-COVID-19 functional status				<0.001
No functional limitations	82 (39%)	76 (78%)	6 (5%)	
Negligible functional limitations	21 (10%)	11 (11%)	10 (9%)	
Slight functional limitations	54 (26%)	8 (8%)	46 (41%)	
Moderate functional limitations	41 (20%)	2 (2%)	39 (35%)	
Severe functional limitations	6 (3%)	0 (0%)	6 (5%)	
Missing	4 (2%)	0 (0%)	4 (4%)	

Continuous variables presented as median (IQR) and compared using the Kruskal-Wallis test; categorical and binary variables presented as n(%) and compared using the Pearson χ2 test (or Fisher exact test if *n* < 5).

^a^Complaints consist of persistent severe fatigue and difficulty concentrating. ^b^Participants were considered of non-Dutch ethnic origin if they were born outside the Netherlands and at least one parent was born outside the Netherlands; or they were born in the Netherlands but both parents were born outside the Netherlands. ^c^Comorbidities include cardiovascular disease, chronic pulmonary disease, diabetes, neurological disease, haematological disease, rheumatic disease, thyroid disorders, renal disease, liver disease, cancer, and psychiatric illness.

Abbreviations: CFQ, Cognitive Failure Questionnaire; COVID-19, coronavirus disease 2019; HADS, The Hospital Anxiety and Depression Scale; ICU, Intensive Care Unit; ISI, Insomnia Severity Index; PSQI, Pittsburgh Sleep Quality Index.

### Differences in neuropsychological test scores between individuals with and without persistent complaints

**Figure 1.**
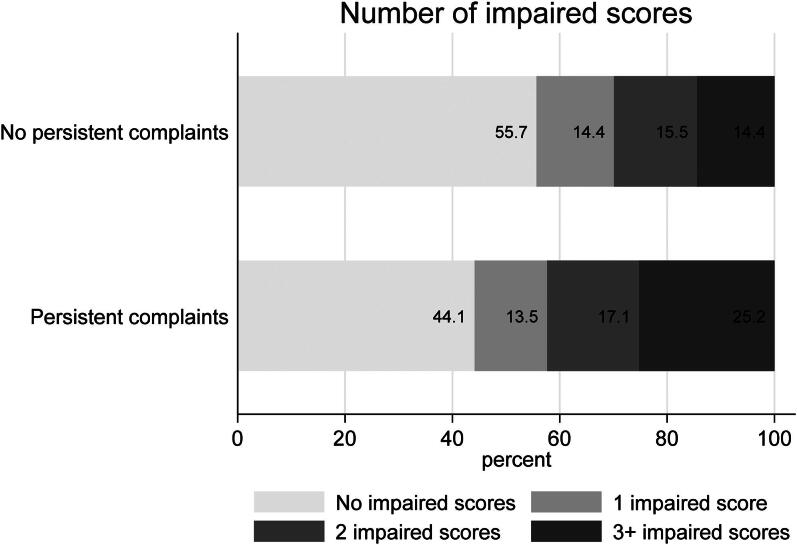
Number of impaired scores out of 15 neuropsychological tests^a^. ^a^Neuropsychological tests: Animal fluency, Controlled Oral Word Association, D2 concentration performance, percentage of errors and total number of items processed, digit-span forward and backward, Rey Auditory Verbal Learning test immediate and recall, Stroop word reading, colour naming and Colour-Word Test, Trail Making Test A, B and B|A. A score was classified as “impaired” below -1.5 SD, adjusted for age, sex and education using population normative data (T = 36). Complaints consist of persistent severe fatigue (CIS fatigue ≥ 35) and difficulty concentrating (CIS concentration ≥ 18).

On the cognitive screening MoCA we found no differences between individuals with (*M* = 27.0, SD = 2.0) and without (*M* = 27.1, SD = 2.2) persistent fatigue and difficulty concentrating when adjusting for age, sex, and educational level (F(4,203)=8.76, *p* < 0.001, R2 = 0.13; β=-0.08, *p* = 0.241). Half of all participants (104/208) had at least one impaired neuropsychological test score, out of 15 tests, and 37% (76/208) had 2 or more impaired scores (Figure 1). The number of impaired test scores did not significantly differ between the group with and without persistent symptoms (χ2(1)=3.77, *p* = 0.052).

Table 2 shows the percentages of participants with and without persistent symptoms scoring *T* ≤ 35 and mean T-scores in both groups for each neuropsychological test. For both groups, the prevalence of impaired functioning was highest on the RAVL immediate and delayed recall (19–23%) and lowest on the animal fluency (1–2%) (Table 2). Using bivariate logistic regression, persistent complaints were associated with slower information processing speed on the Stroop word reading (8% vs. 18%; OR = 2.45, 95%CI = 1.02–5.84), but not on other tests of information processing speed (Table 2). There were no other significant differences in neuropsychological test scores between individuals with versus without persistent complaints in the other cognitive domains (Tables 2 and 3). With linear regression techniques, persistent complaints were associated with lower t-scores on the D2 CP, TMT B, and TMT B|A (Supplementary Table 2).

**Table 2.** Neuropsychological test performance in individuals with and without persistent symptoms, logistic regression.

		Without persistent complaints^a^ *N* = 97		With persistent complaints^a^ *N* = 111		Odds ratios of impaired neuropsychological test scores in participants with persistent complaints^b^		
		Mean score (SD)	N (%) impaired	Mean score (SD)	N (%) impaired	OR	95% CI OR	*p* Value
Global functioning	MoCA total score^c^	27.0 (2.0)		27.1 (2.2)				
Attention (T-score)	D2 CP	54.0 (9.9)	4 (4%)	51.1 (9.4)	6 (5%)	1.30	0.36–4.75	0.681
	D2 E%	56.0 (10.3)	2 (2%)	55.0 (11.7)	5 (5%)	2.19	0.42–11.56	0.349
	D2 TN	53.4 (10.1)	5 (5%)	51.0 (10.1)	8 (7%)	1.40	0.44–4.42	0.558
	Digit-span forward	51.3 (9.8)	5 (5%)	50.7 (11.8)	9 (8%)	1.62	0.53–5.02	0.400
Processing speed (T-score)	Stroop word reading	50.1 (10.2)	8 (8%)	48.7 (13.5)	20 (18%)	2.45	1.02–5.84	0.044
	Stroop colour naming	50.3 (9.0)	7 (7%)	47.8 (12.8)	18 (16%)	2.49	0.99–6.24	0.052
	TMT A	55.8 (11.2)	5 (5%)	54.7 (12.3)	7 (6%)	1.24	0.38–4.04	0.723
Executive functioning (T-score)	COWAT	53.7 (11.6)	7 (7%)	52.4 (12.9)	13 (12%)	1.71	0.65–4.46	0.277
	Digit-span backward	53.1 (11.2)	6 (6%)	54.5 (12.8)	6 (5%)	0.87	0.27–2.78	0.270
	Stroop Colour-Word Test	51.0 (9.1)	6 (6%)	48.1 (11.1)	13 (12%)	2.01	0.73–5.52	0.174
	TMT B	55.0 (10.6)	4 (4%)	51.1 (10.7)	10 (9%)	2.28	0.69–7.51	0.177
	TMT B|A	52.3 (9.6)	5 (5%)	48.3 (9.5)	13 (12%)	2.41	0.83–7.04	0.106
Memory (T-score)	RAVL immediate	45.4 (12.1)	21 (22%)	45.9 (11.0)	22 (20%)	0.89	0.46–1.75	0.745
	RAVL recall	45.6 (12.0)	22 (23%)	47.3 (10.9)	21 (19%)	0.80	0.41–1.56	0.504
	CFT recall^c^	23.3 (5.5)		23.0 (6.5)				
Language (T-score)	Animal fluency	55.0 (10.6)	1 (1%)	55.5 (11.3)	2 (2%)	1.76	0.16–19.73	0.646
Visuoconstruction	CFT copy^c^	34.8 (1.8)		34.6 (2.3)				

Variables presented as mean (SD) score and number and percentage (%) impaired test score (*T* < 36).

^a^Complaints consist of persistent severe fatigue (CIS fatigue ≥35) and difficulty concentrating (CIS concentration ≥18). ^b^Logistic regression analysis. ^c^Unadjusted total score.

Abbreviations: CFT, Rey Complex Figure Test; COWAT, Controlled Oral Word Association Test; CP, concentration performance; E%, percentage of errors; MoCA, The Montreal Cognitive Assessment; RAVL, Rey Auditory Verbal Learning test; TMT, Trail Making Test; TN, total number of items processed.

**Table 3.** Neuropsychological test performance in individuals with and without persistent symptoms, linear regression.

	The Montreal Cognitive Assessment					
	Coef.	SE	t	p	95% CI	
Persistent complaints (reference = no persistent complaints)^a^	−0.348	0.296	−1.18	0.241	−0.932	0.235
Age in years	−0.038	0.011	−3.40	0.001	−0.060	−0.016
Sex (reference = male)	0.186	0.308	0.61	0.546	−0.421	0.793
Highest level of education (reference = low)	0.858	0.251	3.41	0.001	0.362	1.353
Constant	26.696	1.283	20.81	0.000	24.166	29.226
	Rey complex figure test copy condition					
	Coef.	SE	t	p	95% CI	
Persistent complaints (reference = no persistent complaints) ^a^	−0.510	0.294	−1.73	0.084	−1.090	0.070
Age in years	−0.033	0.011	−2.96	0.003	−0.054	−0.011
Sex (reference = male)	0.085	0.306	0.28	0.781	−0.518	0.688
Highest level of education (reference = low)	0.978	0.250	3.91	0.000	0.485	1.470
Constant	34.005	1.276	26.65	0.000	31.489	36.521
	Rey complex figure test recall condition					
	Coef.	SE	t	p	95% CI	
Persistent complaints (reference = no persistent complaints)^a^	−0.755	0.840	−0.90	0.370	−2.412	0.902
Age in years	−0.119	0.032	−3.76	0.000	−0.181	−0.056
Sex (reference = male)	−1.796	0.874	−2.05	0.041	−3.520	−0.072
Highest level of education (reference = low)	2.447	0.714	3.43	0.001	1.040	3.854
Constant	26.254	3.644	7.20	0.000	19.069	33.440

^a^Complaints consist of persistent severe fatigue (CIS fatigue ≥35) and difficulty concentrating (CIS concentration ≥18).

When a more or less stringent cutoff for TOMM trial 1 or both TOMM trials are used, results from the logistic regression remained essentially unchanged. There were slight differences with respect to the significance of the Stroop (Supplementary Table 3).

#### Potential confounders of neuropsychological test performance

Potential confounders of neuropsychological test performance were explored using logistic regression techniques for the following neuropsychological tests: COWAT, RAVL immediate, RAVL recall, Stroop word reading, Stroop colour naming, Stroop Colour-Word. Additionally, for all neuropsychological tests potential confounders were explored using linear regressions. Models are presented in Supplementary Tables 4 and 5a–r. Non-Dutch participants were more likely to have lower immediate memory (RAVL)(OR = 2.80, 95%CI = 1.00–7.84) and lower T-scores on the D2 E% (β=-0.17, t(192)=2.34, *p* = 0.02), TMT A (β=-0.28, t(195)=-4.03, *p* < 0.001), TMT B (β=-0.22, t(194)=-3.20, *p* = 0.002), and animal fluency (β=-0.19, t(195)=-2.60, *p* = 0.01). Participants who had three or more comorbidities had lower verbal memory (RAVL immediate, OR = 7.46, 95%CI = 1.64–33.99; RAVL recall, OR = 6.03, 95%CI = 1.33–27.37). Hospital admission for COVID-19 was associated with worse visual memory (CFT copy condition) (β=-0.21, t(202)=-3.09, *p* = 0.02) and slower set switching (TMT-B) (β=-0.14, t(204)=-2.09, *p* = 0.038). Clinically relevant symptoms of depression were associated with slower set switching (TMT-B)(β=-0.16, t(204)=-2.15, *p* = 0.033). Being completely vaccinated at the time of infection was associated with better visual memory (CFT copy condition)(β = 0.15, t(192)=2.14, *p* = 0.033), visuo-construction (CFT recall condition)(β = 0.15, t(192)=2.14, *p* = 0.033) and sustained attention (D2 TN)(β = 0.16, t(192)=2.15, *p* = 0.033). There were no significant associations for psychiatric comorbidity, ApoE-ɛ4 genotype or months since first infection.

## Discussion

In this study comparing participants with and without persistent severe fatigue and difficulty concentrating approximately two years after SARS-CoV-2 infection, participants with persistent complaints had an increased likelihood of an impaired performance on the Stroop word reading, but not on other tests of information processing speed. We found no other significant differences in the prevalence of impaired performance on neuropsychological tests between individuals with and without persistent severe fatigue and difficulty concentrating.

Despite cognitive problems being one of the most commonly reported symptoms among patients with PASC, objective cognitive functioning approximately two years after COVID-19 has not yet been extensively studied (Crivelli et al., 2022). Earlier studies have reported inconsistent associations between fatigue, subjective cognitive complaints, and objective neuropsychological test performance up to 15 months after infection (Schild et al., 2023; Delgado-Alonso et al., 2022; Mancilla-Corona et al., 2023). The present findings are in line with studies among individuals with myalgic encephalomyelitis/chronic fatigue syndrome (ME/CFS), also characterized by severe fatigue, which showed impairment in processing speed in a subset of patients compared to healthy controls, while performance on attention, memory, executive functioning, verbal ability, and visuospatial functions were comparable between both groups (Cockshell & Mathias, 2013). Regardless, we found (minor) differences in one neuropsychological test out of 15 tests and cannot rule out normal variation independent of COVID-19 (Binder et al., 2009).

In the current study, we excluded 22 participants from the analysis due to indications of invalid performance, all of whom had persistent complaints (16.5% (22/133)). Excluding participants with invalid tests performance is expected to improve the validity of cognitive test results of the sample analysed in this study. This percentage is in line with a recent meta-analysis of pooled base rates of performance validity test failure across various clinical patient groups (Roor et al., 2023). It is unclear which TOMM cutoff should be used for post COVID populations. The use of different cutoffs resulted in slight differences in the significance of test results, which can indicate that the results are not stable. In all situations, persistent complaints were only significantly associated with impaired scores on the Stroop (Stroop Colour naming, Word reading or Colour-Word Test depending on the selection of the TOMM cutoff).

In the present study, individuals with persistent complaints more frequently reported depressive symptoms, consistent with previous studies among patients with PASC (Ceban et al., 2022). Also, in conditions such as traumatic brain injury or ME/CFS, subjective cognitive complaints have been linked to higher levels of depressive symptoms (Cockshell & Mathias, 2013; Bell et al., 2023). The role and aetiology of depressive symptoms within PASC is currently unclear, and could either be directly related to pathophysiology, or be secondary to the limiting effects of post infectious symptoms.

As previously described, the risk of PASC was higher among (hospitalized) COVID-19 patients with a migration background than Dutch origin patients (Chilunga et al., 2023), which was also observed in this study. Non-Dutch origin was associated with higher degree of performance invalidity, and worse performance on several neuropsychological tests. It has been described that in the general population there can be a greater risk of false-positive impaired cognitive functioning in migrant groups (Anapa et al., 2021), which could influence the current findings. Speaking Dutch as a second language could be related to performance validity (Lippa, 2018).

Interestingly, we found few effects on the association between persistent complaints and neuropsychological test scores for COVID-19 clinical factors such as hospitalization following acute infection, months since first infection or vaccination status at time of infection. We hypothesized that severe initial COVID-19, requiring hospitalization, would increase the risk of impaired cognitive functioning after COVID-19, as is seen with PASC in general (Zeng et al., 2023), but found this only for visual memory. Some studies have found an association between severity of initial COVID-19 and test performance (Crivelli et al., 2022) however others have not (García-Sánchez et al., 2022). Furthermore, while ApoE-ɛ4 genotype is associated with severe initial COVID-19 disease and an increased risk of future cognitive decline (MacAulay et al., 2020; Safdari Lord et al., 2022), we did not find that neuropsychological test scores were associated with ApoE-ɛ4 carrier status.

There are several strengths to our study. First, the study included participants who had their (first) SARS-CoV-2 infection about two years before the neuropsychological assessment, which has only scarcely been reported on previously. Secondly, we included participants covering the full spectrum of COVID-19 disease severity in the initial phase of infection. Thirdly, we recruited *via* existing prospective cohorts to limit self-referral bias. Nevertheless, as not all those invited chose to participate, our study might still be prone to selection bias. For example, most participants had a relatively high level of education, which is also reported in the original cohorts, and limits the ability to generalize our findings to other educational levels. Fourthly, this study describes functioning of all cognitive domains using commonly used neuropsychological tests. A limitation of this study is that we were unable to adjust for multiple covariates in one logistic regression model due to the limited number of individuals with impaired scores on the neuropsychological tests. In this study a lenient criterion for impaired scores was used (-1.5 SD), suggesting the absence of significant associations is not underestimated. One could, however, state that this criterion is too lenient and leads to overestimating the presence of cognitive impairment. Another limitation is the lack of information on pre-morbid intellectual functioning, which could impact performance on the neuropsychological tests.

In conclusion, the majority of individuals with subjective severe fatigue and difficulty concentrating after COVID-19 did not show substantial and consistent cognitive impairment, but persistent severe fatigue and difficulty concentrating were associated with indications for slower processing speed in a subgroup of patients. These findings implicate that extensive neuropsychological testing may be insensitive in detecting cognitive impairment in individuals with subjective severe fatigue and difficulty concentrating after SARS-CoV-2 infection.

## Supplementary Material

Supplemental Material

## Data Availability

The data collected for this study are available on request *via* the corresponding author up to 15 years after the end of the study, taking legal restrictions into account.

## Presentation at previous meetings

A preliminary version of the results of this paper was presented as an oral presentation at the ECCMID conference in Copenhagen in April 2023 (number O0049). Results were also presented as part of a symposium at the ICBM conference in Vancouver in August 2023 (number 156).

## VeCosCo study group

Frederik Barkhof, Indiana Buenting, Godelieve de Bree, Eva Camper, Irene Csorba, Elsmarieke van de Giessen, Sandeep Golla, Annelotte Kooij, Roos Rikken, Arjen J.C. Slooter, Muska Uriakhel, Anne Visser.
