# Supplementary material for: Neuropsychological functioning after COVID-19: Minor differences between individuals with and without persistent complaints after SARS-CoV-2 infection
Source: Clin Neuropsychol. 2024 Jul 17;39(2):347–62. doi: 10.1080/13854046.2024.2379508 (PMC11875436; doi:10.1080/13854046.2024.2379508)
Supplement: Supplemental Material [file NTCN_A_2379508_SM8266.docx]

# Supplementary information to: Neuropsychological functioning after COVID-19

Minor differences between individuals with and without persistent symptoms after SARS-CoV-2 infection

**Tables**

Supplementary Table 1. Socio-demographic and clinical characteristics of included and excluded participants with persistent complaints.

Supplementary Table 2. Neuropsychological test performance in individuals with and without persistent symptoms, linear regression

Supplementary Table 3. Neuropsychological test performance in individuals with and without persistent symptoms using different TOMM cutoffs, logistic regression

Supplementary Table 4. Potential confounders of the relationship between persistent complaints and neuropsychological test performance, logistic regression

Supplementary Table 5. Potential confounders of the relationship between persistent complaints and neuropsychological test performance, linear regression

# eTable 1. Socio-demographic and clinical characteristics of included and excluded participants with persistent complaints.

|  | **Participants with persistent complaints** | | |
| --- | --- | --- | --- |
|  | **Included** | **Excluded** | **p-value** |
|  | N=111 | N=22 |  |
| Female sex | 79 (71%) | 17 (77%) | 0.56 |
| Age, years | 50.2 (12.4) | 49.9 (9.5) | 0.93 |
| Highest level of education |  |  | 0.57 |
| Low | 4 (4%) | 0 (0%) |  |
| Middle | 28 (25%) | 7 (32%) |  |
| High | 79 (71%) | 15 (68%) |  |
| Non-Dutch origin^a^ | 14 (13%) | 7 (32%) | 0.02 |
| Missing | 9 (8%) | 2 (9%) |  |
| Number of comorbiditiesb |  |  | 0.40 |
| 0 | 59 (53%) | 7 (32%) |  |
| 1 | 33 (30%) | 9 (41%) |  |
| 2 | 12 (11%) | 2 (9%) |  |
| 3 or more | 6 (5%) | 2 (9%) |  |
| Missing | 1 (1%) | 2 (9%) |  |
| Psychiatric comorbidity before COVID-19 | 5 (5%) | 4 (18%) | 0.01 |
| Missing | 3 (3%) | 2 (9%) |  |
| Months since first SARS-CoV-2 infection | 23.0 (17.0-30.0) | 19.5 (14.0-30.0) | 0.14 |
| Hospital admission for COVID-19 | 32 (29%) | 8 (36%) | 0.48 |
| ICU admission | 10 (9%) | 1 (5%) | 0.49 |
| Vaccinated at time of infection | 13 (12%) | 5 (23%) | 0.20 |
| Missing | 5 (5%) | 0 (0%) |  |
| CIS total score | 102.0 (91.0-112.5) | 114.0 (106.0-127.0) | <0.001 |
| HADS total score | 6.0 (3.0-9.0) | 8.0 (6.0-11.5) | 0.01 |
| Clinically relevant symptoms of depression (HADS≥8) | 43 (39%) | 14 (64%) | 0.03 |

Continuous variables presented as median (IQR) and compared using the Kruskal-Wallis test; categorical and binary variables presented as n(%) and compared using the Pearson χ2 test (or Fisher exact test if n<5).

^a^ Participants were considered of non-Dutch ethnic origin if they were born outside the Netherlands and at least one parent was born outside the Netherlands; or they were born in the Netherlands but both parents were born outside the Netherlands. ^b^Comorbidities include cardiovascular disease, chronic pulmonary disease, diabetes, neurological disease, haematological disease, rheumatic disease, thyroid disorders, renal disease, liver disease, cancer, and psychiatric illness.

Abbreviations: CIS, Checklist Individual Strength; COVID-19, coronavirus disease 2019; HADS, The Hospital Anxiety and Depression Scale; ICU, Intensive Care Unit.

# eTable 2. Neuropsychological test performance in individuals with and without persistent symptoms, linear regression

|  | **Coef.** | **SE** | **t** | **p** | **95% CI** | |
| --- | --- | --- | --- | --- | --- | --- |
| **D2 CP** | **-3.049** | **1.355** | **-2.249** | **0.026** | **-5.721** | **-0.376** |
| D2 E% | -1.022 | 1.545 | -0.662 | 0.509 | -4.069 | 2.024 |
| D2 TN | -2.579 | 1.420 | -1.816 | 0.071 | -5.380 | 0.222 |
| Digit-span forward | -0.581 | 1.517 | -0.383 | 0.702 | -3.572 | 2.411 |
| Stroop Word | -0.985 | 1.705 | -0.578 | 0.564 | -4.346 | 2.376 |
| Stroop Color | -2.571 | 1.551 | -1.658 | 0.099 | -5.628 | 0.487 |
| TMT A | -1.123 | 1.638 | -0.686 | 0.494 | -4.353 | 2.106 |
| COWAT | -1.364 | 1.713 | -0.796 | 0.427 | -4.742 | 2.014 |
| Digit-span backward | 1.471 | 1.680 | 0.875 | 0.382 | -1.842 | 4.784 |
| Stroop Color-Word Test | -2.299 | 1.453 | -1.583 | 0.115 | -5.163 | 0.565 |
| **TMT B** | **-3.909** | **1.481** | **-2.639** | **0.009** | **-6.828** | **-0.989** |
| **TMT B\|A** | **-4.032** | **1.326** | **-3.041** | **0.003** | **-6.646** | **-1.418** |
| RAVL immediate | 0.554 | 1.602 | 0.346 | 0.730 | -2.604 | 3.712 |
| RAVL recall | 1.720 | 1.585 | 1.085 | 0.279 | -1.405 | 4.845 |
| Animal fluency | 0.508 | 1.526 | 0.333 | 0.739 | -2.499 | 3.516 |

Abbreviations: COWAT, Controlled Oral Word Association Test; CP, concentration performance; E%, percentage of errors; MoCA, The Montreal Cognitive Assessment; RAVL, Rey Auditory Verbal Learning test; TMT, Trail Making Test; TN, total number of items processed. Significant p-values are in bold.

# eTable 3. Neuropsychological test performance in individuals with and without persistent symptoms, logistic regression

|  | TOMM Trial 1 < 40  N=211 | | TOMM Trial 1 < 43  N=205 | | TOMM Trial 1 < 44  N=203 | | TOMM Trial 1 < 45 & Trial 2 < 45  N=221 | |
| --- | --- | --- | --- | --- | --- | --- | --- | --- |
|  | OR | 95% CI OR | OR | 95% CI OR | OR | 95% CI OR | OR | 95% CI OR |
| D2 CP | 1.502 | 0.426-5.296 | 1.351 | 0.370-4.942 | 1.820 | 0.442-7.490 | 2.459 | 0.767-7.885 |
| D2 E% | 2.153 | 0.408-11.357 | 2.279 | 0.432-12.033 | 2.277 | 0.431-12.024 | 2.806 | 0.569-13.829 |
| D2 TN | 1.558 | 0.504-4.817 | 1.455 | 0.459-4.609 | 1.837 | 0.535-6.308 | 2.500 | 0.875-7.145 |
| Digit-span forward | 1.769 | 0.583-5.366 | 1.275 | 0.391-4.158 | 1.274 | 0.391-4.155 | 2.342 | 0.813-6.746 |
| Stroop word reading | **2.512** | **1.058-5.964** | 2.375 | 0.988-5.707 | 2.375 | 0.988-5.710 | **3.245** | **1.405-7.494** |
| Stroop colour naming | **2.736** | **1.103-6.782** | **2.571** | **1.024-6.457** | **2.571** | **1.024-6.460** | **3.579** | **1.485-8.623** |
| TMT A | 1.389 | 0.439-4.393 | 1.275 | 0.391-4.158 | 1.274 | 0.391-4.155 | 1.791 | 0.601-5.340 |
| COWAT | 1.800 | 0.695-4.659 | 1.759 | 0.672-4.609 | 1.758 | 0.671-4.608 | 1.905 | 0.751-4.833 |
| Digit-span backward | 0.992 | 0.322-3.058 | 0.736 | 0.217-2.493 | 0.735 | 0.217-2.491 | 1.046 | 0.350-3.122 |
| Stroop Colour-Word Test | 2.298 | 0.855-6.176 | 2.075 | 0.757-5.694 | 2.074 | 0.756-5.693 | **3.454** | **1.346-8.860** |
| TMT B | 2.456 | 0.756-7.981 | 2.347 | 0.711-7.745 | 3.162 | 0.843-11.851 | 2.694 | 0.849-8.543 |
| TMT B\|A | 2.548 | 0.883-7.353 | 2.491 | 0.854-7.266 | 3.146 | 0.989-10.008 | 2.696 | 0.951-7.645 |
| RAVL immediate | 0.915 | 0.470-1.779 | 0.926 | 0.472-1.814 | 0.872 | 0.442-1.721 | 0.960 | 0.502-1.836 |
| RAVL recall | 0.770 | 0.394-1.506 | 0.775 | 0.393-1.528 | 0.726 | 0.365-1.444 | 0.776 | 0.403-1.497 |
| Animal fluency | 1.714 | 0.153-19.199 | 1.811 | 0.162-20.294 | 1.810 | 0.161-20.277 | 4.034 | 0.463-35.109 |

Odds ratios of impaired neuropsychological test scores in participants with persistent complaints. Complaints consist of persistent severe fatigue (CIS fatigue ≥35) and difficulty concentrating (CIS concentration ≥18). Significant p-values are in bold.

Abbreviations: COWAT, Controlled Oral Word Association Test; CP, concentration performance; E%, percentage of errors; RAVL, Rey Auditory Verbal Learning test; TMT, Trail Making Test; TN, total number of items processed; TOMM, Test of Memory Malingering.

# eTable 4. Potential confounders of the relationship between persistent complaints and neuropsychological test performance, logistic regression

|  | COWAT | | RAVL immediate | | RAVL recall | |
| --- | --- | --- | --- | --- | --- | --- |
|  | OR | 95% CI | OR | 95% CI | OR | 95% CI |
| Persistent complaints (reference = no) ^a^ | 1.71 | 0.65 - 4.46 | 0.89 | 0.46 - 1.75 | 0.80 | 0.41 - 1.56 |
| Persistent complaints (reference = no) ^a^ | 1.69 | 0.63 - 4.51 | 0.73 | 0.36 - 1.49 | 0.76 | 0.38 - 1.53 |
| Non-Dutch origin ^b^ (reference = Dutch origin) | 2.42 | 0.70 - 8.33 | **2.80** | **1.00 - 7.84** | 1.09 | 0.34 - 3.55 |
| Persistent complaints (reference = no) | 1.80 | 0.68 - 4.78 | 0.81 | 0.40 - 1.63 | 0.70 | 0.35 - 1.41 |
| Number of comorbidities ^c^ (reference= no) |  |  |  |  |  |  |
| 1 comorbidity | 0.32 | 0.09 - 1.18 | 1.06 | 0.47 - 2.39 | 0.64 | 0.28 - 1.78 |
| 2 comorbidities | 0.24 | 0.03 - 1.86 | 1.17 | 0.42 - 3.26 | 0.39 | 0.11 - 1.39 |
| 3 or more comorbidities |  |  | **7.69** | **1.68 - 35.20** | **6.13** | **1.35 - 27.88** |
| Persistent complaints (reference = no) ^a^ |  |  | 0.92 | 0.47 - 1.81 | 0.81 | 0.41 - 1.59 |
| Psychiatric comorbidity (reference = no) |  |  | 2.64 | 0.71 - 9.83 | 0.92 | 0.19 - 4.54 |
| Persistent complaints (reference = no) ^a^ | 1.71 | 0.65 - 4.49 | 0.86 | 0.44 - 1.69 | 0.76 | 0.39 - 1.50 |
| ApoE-ɛ4 genotype (reference = non ɛ4-carriers) | 1.03 | 0.37 - 2.83 | 0.54 | 0.24 - 1.20 | 0.53 | 0.24 - 1.19 |
| Persistent complaints (reference = no) ^a^ | 1.69 | 0.64 - 4.44 | 0.90 | 0.46 - 1.75 | 0.80 | 0.41 - 1.57 |
| Hospital admission for COVID-19 (reference = no) | 0.39 | 0.11 - 1.38 | 1.02 | 0.49 - 2.13 | 1.34 | 0.66 - 2.73 |
| Persistent complaints (reference = no) ^a^ | 1.68 | 0.64 - 4.41 | 0.89 | 0.46 - 1.75 | 0.79 | 0.40 - 1.55 |
| Months since first SARS-CoV-2 infection | 1.01 | 0.95 - 1.08 | 1.00 | 1.00 - 1.00 | 1.00 | 0.96 - 1.05 |
| Persistent complaints (reference = no) ^a^ | 1.86 | 0.69 - 5.03 | 0.94 | 0.47 - 1.91 | 0.78 | 0.38 - 1.57 |
| Vaccinated at time of infection (reference = no) | 1.66 | 0.54 - 5.06 | 1.40 | 0.61 - 3.24 | 0.92 | 0.38 - 2.24 |
| Persistent complaints (reference = no) ^a^ | 1.36 | 0.46 - 4.00 | 0.84 | 0.40 - 1.78 | 0.85 | 0.40 - 1.78 |
| Clinically relevant symptoms of depression (HADS ≥8) | 1.77 | 0.59 - 5.35 | 1.19 | 0.49 - 2.91 | 0.83 | 0.33 - 2.12 |

|  | Stroop Word | | Stroop Color | | Stroop Color-Word | |
| --- | --- | --- | --- | --- | --- | --- |
|  | OR | 95% CI | OR | 95% CI | OR | 95% CI |
| Persistent complaints (reference = no) ^a^ | 2.45 | 1.02 - 5.84 | 2.49 | 0.99 - 6.24 | 2.01 | 0.73 - 5.52 |
| Persistent complaints (reference = no) ^a^ | 2.70 | 1.12 - 6.51 | 2.41 | 0.94 - 6.16 | 1.99 | 0.71 - 5.55 |
| Non-Dutch origin ^b^ (reference = Dutch origin) | 0.93 | 0.25 - 3.50 | 1.76 | 0.52 - 5.97 | 2.52 | 0.73 - 8.74 |
| Persistent complaints (reference = no) | 2.46 | 1.02 - 5.93 | 2.40 | 0.97 - 6.19 | 2.11 | 0.76 - 5.83 |
| Number of comorbidities ^c^ (reference= no) |  |  |  |  |  |  |
| 1 comorbidity | 0.94 | 0.39 - 2.28 | 1.02 | 0.40 - 2.59 | 1.17 | 0.40 - 3.41 |
| 2 comorbidities | 0.21 | 0.03 - 1.64 | 0.26 | 0.03 - 2.06 | 1.33 | 0.37 - 5.25 |
| 3 or more comorbidities |  |  | 0.78 | 0.09 - 6.92 |  |  |
| Persistent complaints (reference = no) ^a^ |  |  | 2.54 | 1.01 - 6.38 |  |  |
| Psychiatric comorbidity (reference = no) |  |  | 0.81 | 0.10 - 6.77 |  |  |
| Persistent complaints (reference = no) ^a^ | 2.41 | 1.01 - 5.76 | 2.44 | 0.97 - 6.14 | 1.92 | 0.70 - 5.30 |
| ApoE-ɛ4 genotype (reference = non ɛ4-carriers) | 0.79 | 0.31 - 1.98 | 0.74 | 0.28 - 1.98 | 0.42 | 0.12 - 1.51 |
| Persistent complaints (reference = no) ^a^ | 2.44 | 1.02 - 5.82 | 2.50 | 0.99 - 6.27 | 2.03 | 0.74 - 5.58 |
| Hospital admission for COVID-19 (reference = no) | 0.77 | 0.31 - 1.93 | 1.15 | 0.46 - 2.86 | 1.45 | 0.54 - 3.90 |
| Persistent complaints (reference = no) ^a^ | 2.44 | 1.02 - 5.84 | 2.46 | 0.98 - 6.19 | 1.98 | 0.72 - 5.45 |
| Months since first SARS-CoV-2 infection | 1.00 | 1.00 - 1.00 | 1.01 | 0.95 - 1.07 | 1.01 | 0.95 - 1.08 |
| Persistent complaints (reference = no) ^a^ | 2.34 | 0.95 - 5.75 | 2.31 | 0.90 - 5.96 | 1.96 | 0.70 - 5.50 |
| Vaccinated at time of infection (reference = no) | 1.13 | 0.39 - 3.32 | 0.98 | 0.30 - 3.14 | 0.88 | 0.24 - 3.28 |
| Persistent complaints (reference = no) ^a^ | 2.44 | 0.96 - 6.23 | 2.08 | 0.76 - 5.70 | 1.59 | 0.52 - 4.90 |
| Clinically relevant symptoms of depression (HADS ≥8) | 1.00 | 0.38 - 2.63 | 1.59 | 0.60 - 0.17 | 1.80 | 0.59 - 5.47 |

^a^ Complaints consist of persistent severe fatigue (CIS fatigue ≥35) and concentration problems (CIS concentration ≥18). ^b^ Participants were considered of non-Dutch ethnic origin if they were born outside the Netherlands and at least one parent was born outside the Netherlands; or they were born in the Netherlands but both parents were born outside the Netherlands. ^c^ Comorbidities include cardiovascular disease, chronic pulmonary disease, diabetes, neurological disease, haematological disease, rheumatic disease, thyroid disorders, renal disease, liver disease, cancer, and psychiatric illness. Significant p-values are in bold.

Abbreviations: HADS, The Hospital Anxiety and Depression Scale; COWAT, Controlled Oral Word Association Test; RAVL, Rey Auditory Verbal Learning test; TMT, Trail Making Test.

# eTable 5. Potential confounders of the relationship between persistent complaints and neuropsychological test performance, linear regression

## eTable 5a The Montreal Cognitive Assessment

|  | **Coef.** | **SE** | **t** | **p** | **95% CI** | |
| --- | --- | --- | --- | --- | --- | --- |
| Persistent complaints ^a^ (reference = no) | -0.329 | 0.306 | -1.075 | 0.284 | -0.932 | 0.274 |
| Age in years | -0.039 | 0.011 | -3.401 | 0.001 | -0.061 | -0.016 |
| Sex (reference = male) | 0.235 | 0.319 | 0.737 | 0.462 | -0.394 | 0.863 |
| Highest level of education (reference = low) | 0.806 | 0.258 | 3.129 | 0.002 | 0.298 | 1.315 |
| Non-Dutch origin ^b^ (reference = Dutch origin) | -0.512 | 0.488 | -1.049 | 0.295 | -1.476 | 0.451 |
| Constant | 26.821 | 1.317 | 20.360 | 0.000 | 24.223 | 29.420 |
|  | **Coef.** | **SE** | **t** | **p** | **95% CI** | |
| Persistent complaints ^a^ (reference = no) | -0.311 | 0.298 | -1.042 | 0.299 | -0.899 | 0.277 |
| Age in years | -0.038 | 0.012 | -3.233 | 0.001 | -0.061 | -0.015 |
| Sex (reference = male) | 0.196 | 0.313 | 0.626 | 0.532 | -0.421 | 0.812 |
| Highest level of education (reference = low) | 0.828 | 0.256 | 3.240 | 0.001 | 0.324 | 1.332 |
| Number of comorbidities ^c^ (reference= no) | -0.052 | 0.174 | -0.301 | 0.763 | -0.394 | 0.290 |
| Constant | 26.771 | 1.293 | 20.705 | 0.000 | 24.221 | 29.320 |
|  | **Coef.** | **SE** | **t** | **p** | **95% CI** | |
| Persistent complaints ^a^ (reference = no) | -0.344 | 0.299 | -1.152 | 0.251 | -0.933 | 0.245 |
| Age in years | -0.039 | 0.011 | -3.444 | 0.001 | -0.061 | -0.017 |
| Sex (reference = male) | 0.179 | 0.320 | 0.559 | 0.577 | -0.453 | 0.811 |
| Highest level of education (reference = low) | 0.823 | 0.253 | 3.260 | 0.001 | 0.325 | 1.322 |
| Psychiatric comorbidity (reference= no) | -0.115 | 0.658 | -0.175 | 0.861 | -1.412 | 1.182 |
| Constant | 26.836 | 1.300 | 20.637 | 0.000 | 24.272 | 29.401 |
|  | **Coef.** | **SE** | **t** | **p** | **95% CI** | |
| Persistent complaints ^a^ (reference = no) | -0.312 | 0.297 | -1.052 | 0.294 | -0.897 | 0.273 |
| Age in years | -0.037 | 0.011 | -3.344 | 0.001 | -0.059 | -0.015 |
| Sex (reference = male) | 0.172 | 0.308 | 0.559 | 0.576 | -0.434 | 0.778 |
| Highest level of education (reference = low) | 0.859 | 0.251 | 3.424 | 0.001 | 0.364 | 1.354 |
| ApoE-ɛ4 genotype (reference = non ɛ4-carriers) | 0.377 | 0.298 | 1.264 | 0.208 | -0.211 | 0.965 |
| Constant | 26.550 | 1.286 | 20.637 | 0.000 | 24.013 | 29.086 |
|  | **Coef.** | **SE** | **t** | **p** | **95% CI** | |
| Persistent complaints ^a^ (reference = no) | -0.329 | 0.298 | -1.105 | 0.270 | -0.917 | 0.258 |
| Age in years | -0.036 | 0.012 | -3.098 | 0.002 | -0.059 | -0.013 |
| Sex (reference = male) | 0.167 | 0.310 | 0.538 | 0.591 | -0.444 | 0.778 |
| Highest level of education (reference = low) | 0.836 | 0.254 | 3.287 | 0.001 | 0.334 | 1.337 |
| Hospital admission for COVID-19 (reference = no) | -0.201 | 0.323 | -0.622 | 0.535 | -0.838 | 0.436 |
| Constant | 26.731 | 1.286 | 20.782 | 0.000 | 24.195 | 29.268 |
|  | **Coef.** | **SE** | **t** | **p** | **95% CI** | |
| Persistent complaints ^a^ (reference = no) | -0.334 | 0.298 | -1.123 | 0.263 | -0.922 | 0.253 |
| Age in years | -0.037 | 0.011 | -3.329 | 0.001 | -0.059 | -0.015 |
| Sex (reference = male) | 0.150 | 0.308 | 0.488 | 0.626 | -0.458 | 0.758 |
| Highest level of education (reference = low) | 0.839 | 0.251 | 3.339 | 0.001 | 0.343 | 1.334 |
| Months since first SARS-CoV-2 infection | -0.000 | 0.000 | -0.937 | 0.350 | -0.000 | 0.000 |
| Constant | 26.742 | 1.283 | 20.843 | 0.000 | 24.212 | 29.272 |
|  | **Coef.** | **SE** | **t** | **p** | **95% CI** | |
| Persistent complaints ^a^ (reference = no) | -0.225 | 0.306 | -0.736 | 0.463 | -0.830 | 0.379 |
| Age in years | -0.039 | 0.012 | -3.348 | 0.001 | -0.061 | -0.016 |
| Sex (reference = male) | 0.258 | 0.316 | 0.816 | 0.416 | -0.366 | 0.882 |
| Highest level of education (reference = low) | 0.816 | 0.265 | 3.074 | 0.002 | 0.292 | 1.340 |
| Vaccinated at time of infection (reference = no) | 0.567 | 0.367 | 1.543 | 0.125 | -0.158 | 1.291 |
| Constant | 26.575 | 1.334 | 19.926 | 0.000 | 23.944 | 29.205 |
|  | **Coef.** | **SE** | **t** | **p** | **95% CI** | |
| Persistent complaints ^a^ (reference = no) | -0.182 | 0.319 | -0.572 | 0.568 | -0.812 | 0.447 |
| Age in years | -0.038 | 0.011 | -3.471 | 0.001 | -0.060 | -0.017 |
| Sex (reference = male) | 0.205 | 0.307 | 0.666 | 0.506 | -0.402 | 0.811 |
| Highest level of education (reference = low) | 0.876 | 0.251 | 3.487 | 0.001 | 0.381 | 1.371 |
| Clinically relevant symptoms of depression (HADS ≥8) | -0.501 | 0.365 | -1.372 | 0.172 | -1.221 | 0.219 |
| Constant | 26.686 | 1.280 | 20.843 | 0.000 | 24.162 | 29.211 |

## eTable 5b D2 Concentration Performance

|  | **Coef.** | **SE** | **t** | **p** | **95% CI** | |
| --- | --- | --- | --- | --- | --- | --- |
| Persistent complaints ^a^ (reference = no) | -2.787 | 1.418 | -1.966 | 0.051 | -5.584 | 0.009 |
| Non-Dutch origin ^b^ (reference = Dutch origin) | -3.903 | 2.389 | -1.634 | 0.104 | -8.615 | 0.809 |
| Constant | 54.378 | 1.018 | 53.428 | 0.000 | 52.370 | 56.385 |
|  | **Coef.** | **SE** | **t** | **p** | **95% CI** | |
| Persistent complaints ^a^ (reference = no) | -3.083 | 1.371 | -2.250 | 0.026 | -5.786 | -0.381 |
| Number of comorbidities ^c^ (reference= no) | 0.652 | 0.803 | 0.812 | 0.418 | -0.931 | 2.234 |
| Constant | 53.754 | 1.127 | 47.696 | 0.000 | 51.532 | 55.977 |
|  | **Coef.** | **SE** | **t** | **p** | **95% CI** | |
| Persistent complaints ^a^ (reference = no) | -3.176 | 1.380 | -2.302 | 0.022 | -5.897 | -0.455 |
| Psychiatric comorbidity (reference= no) | 2.025 | 3.166 | 0.640 | 0.523 | -4.219 | 8.269 |
| Constant | 54.062 | 1.021 | 52.972 | 0.000 | 52.050 | 56.075 |
|  | **Coef.** | **SE** | **t** | **p** | **95% CI** | |
| Persistent complaints ^a^ (reference = no) | -3.713 | 1.413 | -2.628 | 0.009 | -6.499 | -0.926 |
| ApoE-ɛ4 genotype (reference = non ɛ4-carriers) | 1.913 | 1.505 | 1.271 | 0.205 | -1.055 | 4.882 |
| Constant | 53.964 | 1.163 | 46.387 | 0.000 | 51.669 | 56.259 |
|  | **Coef.** | **SE** | **t** | **p** | **95% CI** | |
| Persistent complaints ^a^ (reference = no) | -3.087 | 1.355 | -2.278 | 0.024 | -5.759 | -0.416 |
| Hospital admission for COVID-19 (reference = no) | -1.650 | 1.485 | -1.111 | 0.268 | -4.578 | 1.279 |
| Constant | 54.661 | 1.091 | 50.104 | 0.000 | 52.510 | 56.813 |
|  | **Coef.** | **SE** | **t** | **p** | **95% CI** | |
| Persistent complaints ^a^ (reference = no) | 1.367 | -2.202 | 0.029 | -5.708 | -0.315 |  |
| Months since first SARS-CoV-2 infection | -0.001 | 0.001 | -0.615 | 0.539 | -0.002 | 0.001 |
| Constant | 54.106 | 1.000 | 54.094 | 0.000 | 52.134 | 56.078 |
|  | **Coef.** | **SE** | **t** | **p** | **95% CI** | |
| Persistent complaints ^a^ (reference = no) | -1.911 | 1.405 | -1.360 | 0.175 | -4.682 | 0.860 |
| Vaccinated at time of infection (reference = no) | 3.476 | 1.768 | 1.966 | 0.051 | -0.011 | 6.963 |
| Constant | 52.757 | 1.124 | 46.926 | 0.000 | 50.539 | 54.974 |
|  | **Coef.** | **SE** | **t** | **p** | **95% CI** | |
| Persistent complaints ^a^ (reference = no) | -2.314 | 1.507 | -1.535 | 0.126 | -5.285 | 0.658 |
| Clinically relevant symptoms of depression (HADS ≥8) | -2.038 | 1.830 | -1.113 | 0.267 | -5.647 | 1.571 |
| Constant | 54.201 | 0.993 | 54.581 | 0.000 | 52.243 | 56.159 |

## eTable 5c D2 percentage of errors

|  | **Coef.** | **SE** | **t** | **p** | **95% CI** | |
| --- | --- | --- | --- | --- | --- | --- |
| Persistent complaints ^a^ (reference = no) | -0.596 | 1.539 | -0.387 | 0.699 | -3.632 | 2.440 |
| **Non-Dutch origin ^b^ (reference = Dutch origin)** | **-6.063** | **2.593** | **-2.338** | **0.020** | **-11.178** | **-0.948** |
| Constant | 56.397 | 1.105 | 51.048 | 0.000 | 54.218 | 58.576 |
|  | **Coef.** | **SE** | **t** | **p** | **95% CI** | |
| Persistent complaints ^a^ (reference = no) | -1.198 | 1.557 | -0.769 | 0.443 | -4.268 | 1.873 |
| Number of comorbidities ^c^ (reference= no) | -0.108 | 0.912 | -0.118 | 0.906 | -1.906 | 1.690 |
| Constant | 56.143 | 1.280 | 43.846 | 0.000 | 53.618 | 58.668 |
|  | **Coef.** | **SE** | **t** | **p** | **95% CI** | |
| Persistent complaints ^a^ (reference = no) | -1.341 | 1.562 | -0.858 | 0.392 | -4.422 | 1.740 |
| Psychiatric comorbidity (reference= no) | -2.222 | 3.585 | -0.620 | 0.536 | -9.292 | 4.848 |
| Constant | 56.193 | 1.156 | 48.626 | 0.000 | 53.914 | 58.472 |
|  | **Coef.** | **SE** | **t** | **p** | **95% CI** | |
| Persistent complaints ^a^ (reference = no) | -1.256 | 1.604 | -0.783 | 0.435 | -4.421 | 1.909 |
| ApoE-ɛ4 genotype (reference = non ɛ4-carriers) | 0.830 | 1.709 | 0.486 | 0.628 | -2.542 | 4.202 |
| Constant | 55.600 | 1.321 | 42.081 | 0.000 | 52.994 | 58.207 |
|  | **Coef.** | **SE** | **t** | **p** | **95% CI** | |
| Persistent complaints ^a^ (reference = no) | -1.059 | 1.546 | -0.685 | 0.494 | -4.108 | 1.990 |
| Hospital admission for COVID-19 (reference = no) | -1.561 | 1.694 | -0.921 | 0.358 | -4.902 | 1.780 |
| Constant | 56.508 | 1.245 | 45.397 | 0.000 | 54.054 | 58.962 |
|  | **Coef.** | **SE** | **t** | **p** | **95% CI** | |
| Persistent complaints ^a^ (reference = no) | -0.954 | 1.552 | -0.615 | 0.539 | -4.014 | 2.105 |
| Months since first SARS-CoV-2 infection | -0.001 | 0.001 | -1.280 | 0.202 | -0.003 | 0.001 |
| Constant | 55.909 | 1.135 | 49.261 | 0.000 | 53.671 | 58.147 |
|  | **Coef.** | **SE** | **t** | **p** | **95% CI** | |
| Persistent complaints ^a^ (reference = no) | -0.212 | 1.613 | -0.132 | 0.895 | -3.394 | 2.970 |
| Vaccinated at time of infection (reference = no) | 1.425 | 2.030 | 0.702 | 0.484 | -2.579 | 5.430 |
| Constant | 55.226 | 1.291 | 42.777 | 0.000 | 52.680 | 57.773 |
|  | **Coef.** | **SE** | **t** | **p** | **95% CI** | |
| Persistent complaints ^a^ (reference = no) | -1.030 | 1.723 | -0.598 | 0.551 | -4.427 | 2.368 |
| Clinically relevant symptoms of depression (HADS ≥8) | 0.020 | 2.093 | 0.009 | 0.992 | -4.107 | 4.146 |
| Constant | 56.031 | 1.135 | 49.349 | 0.000 | 53.792 | 58.270 |

## eTable 5d D2 total number of items processed

|  | **Coef.** | **SE** | **t** | **p** | **95% CI** | |
| --- | --- | --- | --- | --- | --- | --- |
| Persistent complaints ^a^ (reference = no) | -2.479 | 1.478 | -1.678 | 0.095 | -5.394 | 0.436 |
| Non-Dutch origin ^b^ (reference = Dutch origin) | -2.869 | 2.490 | -1.152 | 0.251 | -7.780 | 2.043 |
| Constant | 53.748 | 1.061 | 50.666 | 0.000 | 51.656 | 55.841 |
|  | **Coef.** | **SE** | **t** | **p** | **95% CI** | |
| Persistent complaints ^a^ (reference = no) | -2.551 | 1.437 | -1.775 | 0.077 | -5.385 | 0.283 |
| Number of comorbidities ^c^ (reference= no) | 0.212 | 0.841 | 0.251 | 0.802 | -1.448 | 1.871 |
| Constant | 53.461 | 1.182 | 45.238 | 0.000 | 51.130 | 55.791 |
|  | **Coef.** | **SE** | **t** | **p** | **95% CI** | |
| Persistent complaints ^a^ (reference = no) | -2.641 | 1.446 | -1.826 | 0.069 | -5.493 | 0.211 |
| Psychiatric comorbidity (reference= no) | 1.716 | 3.319 | 0.517 | 0.606 | -4.829 | 8.261 |
| Constant | 53.504 | 1.070 | 50.014 | 0.000 | 51.395 | 55.614 |
|  | **Coef.** | **SE** | **t** | **p** | **95% CI** | |
| Persistent complaints ^a^ (reference = no) | -2.907 | 1.473 | -1.973 | 0.050 | -5.814 | -0.001 |
| ApoE-ɛ4 genotype (reference = non ɛ4-carriers) | 1.601 | 1.570 | 1.020 | 0.309 | -1.495 | 4.698 |
| Constant | 53.289 | 1.213 | 43.923 | 0.000 | 50.896 | 55.683 |
|  | **Coef.** | **SE** | **t** | **p** | **95% CI** | |
| Persistent complaints ^a^ (reference = no) | -2.623 | 1.419 | -1.848 | 0.066 | -5.421 | 0.176 |
| Hospital admission for COVID-19 (reference = no) | -1.858 | 1.556 | -1.194 | 0.234 | -4.925 | 1.210 |
| Constant | 54.146 | 1.143 | 47.384 | 0.000 | 51.893 | 56.399 |
|  | **Coef.** | **SE** | **t** | **p** | **95% CI** | |
| Persistent complaints ^a^ (reference = no) | -2.521 | 1.434 | -1.758 | 0.080 | -5.348 | 0.306 |
| Months since first SARS-CoV-2 infection | -0.000 | 0.001 | -0.449 | 0.654 | -0.002 | 0.001 |
| Constant | 53.539 | 1.049 | 51.047 | 0.000 | 51.471 | 55.607 |
|  | **Coef.** | **SE** | **t** | **p** | **95% CI** | |
| Persistent complaints ^a^ (reference = no) | -1.582 | 1.461 | -1.083 | 0.280 | -4.465 | 1.300 |
| **Vaccinated at time of infection (reference = no)** | **3.951** | **1.839** | **2.148** | **0.033** | **0.323** | **7.579** |
| Constant | 52.169 | 1.170 | 44.604 | 0.000 | 49.862 | 54.476 |
|  | **Coef.** | **SE** | **t** | **p** | **95% CI** | |
| Persistent complaints ^a^ (reference = no) | -1.621 | 1.577 | -1.028 | 0.305 | -4.729 | 1.488 |
| Clinically relevant symptoms of depression (HADS ≥8) | -2.656 | 1.915 | -1.387 | 0.167 | -6.432 | 1.120 |
| Constant | 53.635 | 1.039 | 51.627 | 0.000 | 51.586 | 55.683 |

## eTable 5e Digit-span forward

|  | **Coef.** | **SE** | **t** | **p** | **95% CI** | |
| --- | --- | --- | --- | --- | --- | --- |
| Persistent complaints ^a^ (reference = no) | -1.402 | 1.529 | -0.917 | 0.360 | -4.417 | 1.613 |
| Non-Dutch origin ^b^ (reference = Dutch origin) | 0.449 | 2.594 | 0.173 | 0.863 | -4.667 | 5.565 |
| Constant | 51.320 | 1.094 | 46.907 | 0.000 | 49.163 | 53.478 |
|  | **Coef.** | **SE** | **t** | **p** | **95% CI** | |
| Persistent complaints ^a^ (reference = no) | -0.758 | 1.527 | -0.496 | 0.620 | -3.768 | 2.252 |
| Number of comorbidities ^c^ (reference= no) | -0.113 | 0.893 | -0.127 | 0.899 | -1.873 | 1.647 |
| Constant | 51.417 | 1.256 | 40.951 | 0.000 | 48.941 | 53.892 |
|  | **Coef.** | **SE** | **t** | **p** | **95% CI** | |
| Persistent complaints ^a^ (reference = no) | -1.115 | 1.510 | -0.738 | 0.461 | -4.092 | 1.863 |
| Psychiatric comorbidity (reference= no) | -0.408 | 3.491 | -0.117 | 0.907 | -7.292 | 6.476 |
| Constant | 51.365 | 1.114 | 46.123 | 0.000 | 49.169 | 53.561 |
|  | **Coef.** | **SE** | **t** | **p** | **95% CI** | |
| Persistent complaints ^a^ (reference = no) | -0.730 | 1.610 | -0.453 | 0.651 | -3.906 | 2.446 |
| ApoE-ɛ4 genotype (reference = non ɛ4-carriers) | 2.118 | 1.713 | 1.236 | 0.218 | -1.261 | 5.496 |
| Constant | 50.579 | 1.330 | 38.029 | 0.000 | 47.956 | 53.203 |
|  | **Coef.** | **SE** | **t** | **p** | **95% CI** | |
| Persistent complaints ^a^ (reference = no) | -0.576 | 1.521 | -0.379 | 0.705 | -3.576 | 2.423 |
| Hospital admission for COVID-19 (reference = no) | 0.225 | 1.659 | 0.135 | 0.892 | -3.047 | 3.496 |
| Constant | 51.250 | 1.224 | 41.876 | 0.000 | 48.837 | 53.663 |
|  | **Coef.** | **SE** | **t** | **p** | **95% CI** | |
| Persistent complaints ^a^ (reference = no) | -0.533 | 1.526 | -0.350 | 0.727 | -3.542 | 2.475 |
| Months since first SARS-CoV-2 infection | -0.001 | 0.001 | -1.072 | 0.285 | -0.003 | 0.001 |
| Constant | 51.220 | 1.113 | 46.019 | 0.000 | 49.026 | 53.415 |
|  | **Coef.** | **SE** | **t** | **p** | **95% CI** | |
| Persistent complaints ^a^ (reference = no) | -0.856 | 1.585 | -0.540 | 0.590 | -3.982 | 2.270 |
| Vaccinated at time of infection (reference = no) | -1.256 | 1.988 | -0.632 | 0.528 | -5.177 | 2.664 |
| Constant | 52.029 | 1.268 | 41.048 | 0.000 | 49.529 | 54.529 |
|  | **Coef.** | **SE** | **t** | **p** | **95% CI** | |
| Persistent complaints ^a^ (reference = no) | -0.433 | 1.698 | -0.255 | 0.799 | -3.780 | 2.914 |
| Clinically relevant symptoms of depression (HADS ≥8) | -0.403 | 2.057 | -0.196 | 0.845 | -4.458 | 3.652 |
| Constant | 51.328 | 1.112 | 46.165 | 0.000 | 49.136 | 53.520 |

## eTable 5f Stroop Word

|  | **Coef.** | **SE** | **t** | **p** | **95% CI** | |
| --- | --- | --- | --- | --- | --- | --- |
| Persistent complaints ^a^ (reference = no) | -1.609 | 1.761 | -0.914 | 0.362 | -5.082 | 1.863 |
| Non-Dutch origin ^b^ (reference = Dutch origin) | -1.832 | 2.988 | -0.613 | 0.540 | -7.725 | 4.060 |
| Constant | 49.439 | 1.260 | 39.234 | 0.000 | 46.954 | 51.924 |
|  | **Coef.** | **SE** | **t** | **p** | **95% CI** | |
| Persistent complaints ^a^ (reference = no) | -1.102 | 1.720 | -0.641 | 0.522 | -4.494 | 2.289 |
| Number of comorbidities ^c^ (reference= no) | 1.118 | 1.006 | 1.112 | 0.268 | -0.865 | 3.102 |
| Constant | 48.621 | 1.415 | 34.370 | 0.000 | 45.832 | 51.411 |
|  | **Coef.** | **SE** | **t** | **p** | **95% CI** | |
| Persistent complaints ^a^ (reference = no) | -1.450 | 1.713 | -0.847 | 0.398 | -4.827 | 1.927 |
| Psychiatric comorbidity (reference= no) | 0.824 | 3.959 | 0.208 | 0.835 | -6.982 | 8.631 |
| Constant | 49.301 | 1.263 | 39.039 | 0.000 | 46.811 | 51.791 |
|  | **Coef.** | **SE** | **t** | **p** | **95% CI** | |
| Persistent complaints ^a^ (reference = no) | -1.512 | 1.781 | -0.849 | 0.397 | -5.025 | 2.001 |
| ApoE-ɛ4 genotype (reference = non ɛ4-carriers) | 2.689 | 1.895 | 1.419 | 0.158 | -1.049 | 6.426 |
| Constant | 48.603 | 1.471 | 33.036 | 0.000 | 45.701 | 51.505 |
|  | **Coef.** | **SE** | **t** | **p** | **95% CI** | |
| Persistent complaints ^a^ (reference = no) | -1.014 | 1.707 | -0.594 | 0.553 | -4.380 | 2.352 |
| Hospital admission for COVID-19 (reference = no) | -1.395 | 1.862 | -0.749 | 0.455 | -5.065 | 2.276 |
| Constant | 49.741 | 1.373 | 36.219 | 0.000 | 47.033 | 52.448 |
|  | **Coef.** | **SE** | **t** | **p** | **95% CI** | |
| Persistent complaints ^a^ (reference = no) | -0.942 | 1.721 | -0.547 | 0.585 | -4.336 | 2.452 |
| Months since first SARS-CoV-2 infection | -0.000 | 0.001 | -0.311 | 0.756 | -0.002 | 0.002 |
| Constant | 49.277 | 1.256 | 39.246 | 0.000 | 46.801 | 51.752 |
|  | **Coef.** | **SE** | **t** | **p** | **95% CI** | |
| Persistent complaints ^a^ (reference = no) | -0.569 | 1.802 | -0.316 | 0.752 | -4.122 | 2.984 |
| Vaccinated at time of infection (reference = no) | 2.772 | 2.259 | 1.227 | 0.221 | -1.684 | 7.228 |
| Constant | 48.597 | 1.441 | 33.733 | 0.000 | 45.756 | 51.438 |
|  | **Coef.** | **SE** | **t** | **p** | **95% CI** | |
| Persistent complaints ^a^ (reference = no) | 0.033 | 1.901 | 0.017 | 0.986 | -3.715 | 3.781 |
| Clinically relevant symptoms of depression (HADS ≥8) | -2.776 | 2.303 | -1.205 | 0.229 | -7.317 | 1.765 |
| Constant | 49.367 | 1.245 | 39.652 | 0.000 | 46.912 | 51.821 |

## eTable 5g Stroop Color

|  | **Coef.** | **SE** | **t** | **p** | **95% CI** | |
| --- | --- | --- | --- | --- | --- | --- |
| Persistent complaints ^a^ (reference = no) | -2.867 | 1.616 | -1.774 | 0.078 | -6.055 | 0.320 |
| Non-Dutch origin ^b^ (reference = Dutch origin) | -2.153 | 2.742 | -0.785 | 0.433 | -7.561 | 3.255 |
| Constant | 50.477 | 1.157 | 43.644 | 0.000 | 48.196 | 52.758 |
|  | **Coef.** | **SE** | **t** | **p** | **95% CI** | |
| Persistent complaints ^a^ (reference = no) | -2.742 | 1.561 | -1.756 | 0.081 | -5.820 | 0.336 |
| Number of comorbidities ^c^ (reference= no) | 1.132 | 0.913 | 1.240 | 0.216 | -0.668 | 2.932 |
| Constant | 49.633 | 1.284 | 38.657 | 0.000 | 47.102 | 52.165 |
|  | **Coef.** | **SE** | **t** | **p** | **95% CI** | |
| Persistent complaints ^a^ (reference = no) | -2.931 | 1.571 | -1.865 | 0.064 | -6.029 | 0.168 |
| Psychiatric comorbidity (reference= no) | -1.793 | 3.633 | -0.493 | 0.622 | -8.956 | 5.371 |
| Constant | 50.458 | 1.159 | 43.541 | 0.000 | 48.173 | 52.743 |
|  | **Coef.** | **SE** | **t** | **p** | **95% CI** | |
| Persistent complaints ^a^ (reference = no) | -3.158 | 1.600 | -1.973 | 0.050 | -6.314 | -0.001 |
| ApoE-ɛ4 genotype (reference = non ɛ4-carriers) | -0.297 | 1.702 | -0.175 | 0.861 | -3.656 | 3.061 |
| Constant | 50.642 | 1.322 | 38.309 | 0.000 | 48.035 | 53.250 |
|  | **Coef.** | **SE** | **t** | **p** | **95% CI** | |
| Persistent complaints ^a^ (reference = no) | -2.563 | 1.555 | -1.648 | 0.101 | -5.628 | 0.502 |
| Hospital admission for COVID-19 (reference = no) | 0.370 | 1.696 | 0.218 | 0.828 | -2.973 | 3.713 |
| Constant | 50.195 | 1.251 | 40.135 | 0.000 | 47.729 | 52.661 |
|  | **Coef.** | **SE** | **t** | **p** | **95% CI** | |
| Persistent complaints ^a^ (reference = no) | -2.488 | 1.565 | -1.590 | 0.113 | -5.573 | 0.596 |
| Months since first SARS-CoV-2 infection | -0.000 | 0.001 | -0.255 | 0.799 | -0.002 | 0.002 |
| Constant | 50.285 | 1.141 | 44.058 | 0.000 | 48.035 | 52.535 |
|  | **Coef.** | **SE** | **t** | **p** | **95% CI** | |
| Persistent complaints ^a^ (reference = no) | -1.635 | 1.640 | -0.997 | 0.320 | -4.870 | 1.600 |
| Vaccinated at time of infection (reference = no) | 3.535 | 2.057 | 1.719 | 0.087 | -0.522 | 7.592 |
| Constant | 49.164 | 1.312 | 37.485 | 0.000 | 46.577 | 51.751 |
|  | **Coef.** | **SE** | **t** | **p** | **95% CI** | |
| Persistent complaints ^a^ (reference = no) | -1.231 | 1.722 | -0.715 | 0.476 | -4.626 | 2.165 |
| Clinically relevant symptoms of depression (HADS ≥8) | -3.653 | 2.087 | -1.751 | 0.082 | -7.767 | 0.461 |
| Constant | 50.385 | 1.128 | 44.669 | 0.000 | 48.161 | 52.608 |

## eTable 5h Trail Making Test a

|  | **Coef.** | **SE** | **t** | **p** | **95% CI** | |
| --- | --- | --- | --- | --- | --- | --- |
| Persistent complaints ^a^ (reference = no) | -0.359 | 1.640 | -0.219 | 0.827 | -3.593 | 2.874 |
| **Non-Dutch origin ^b^ (reference = Dutch origin)** | **-11.205** | **2.782** | **-4.027** | **0.000** | **-16.692** | **-5.718** |
| Constant | 56.417 | 1.173 | 48.077 | 0.000 | 54.103 | 58.731 |
|  | **Coef.** | **SE** | **t** | **p** | **95% CI** | |
| Persistent complaints ^a^ (reference = no) | -1.099 | 1.641 | -0.670 | 0.504 | -4.335 | 2.137 |
| Number of comorbidities ^c^ (reference= no) | 1.831 | 0.960 | 1.908 | 0.058 | -0.061 | 3.724 |
| Constant | 54.651 | 1.350 | 40.486 | 0.000 | 51.989 | 57.312 |
|  | **Coef.** | **SE** | **t** | **p** | **95% CI** | |
| Persistent complaints ^a^ (reference = no) | -1.105 | 1.669 | -0.662 | 0.509 | -4.397 | 2.187 |
| Psychiatric comorbidity (reference= no) | -0.613 | 3.859 | -0.159 | 0.874 | -8.223 | 6.998 |
| Constant | 55.865 | 1.231 | 45.377 | 0.000 | 53.438 | 58.293 |
|  | **Coef.** | **SE** | **t** | **p** | **95% CI** | |
| Persistent complaints ^a^ (reference = no) | -1.507 | 1.743 | -0.865 | 0.388 | -4.946 | 1.931 |
| ApoE-ɛ4 genotype (reference = non ɛ4-carriers) | 1.216 | 1.855 | 0.656 | 0.513 | -2.442 | 4.874 |
| Constant | 55.454 | 1.440 | 38.509 | 0.000 | 52.614 | 58.295 |
|  | **Coef.** | **SE** | **t** | **p** | **95% CI** | |
| Persistent complaints ^a^ (reference = no) | -1.166 | 1.638 | -0.712 | 0.477 | -4.394 | 2.063 |
| Hospital admission for COVID-19 (reference = no) | -2.019 | 1.786 | -1.131 | 0.260 | -5.540 | 1.502 |
| Constant | 56.460 | 1.317 | 42.859 | 0.000 | 53.862 | 59.057 |
|  | **Coef.** | **SE** | **t** | **p** | **95% CI** | |
| Persistent complaints ^a^ (reference = no) | -1.153 | 1.651 | -0.698 | 0.486 | -4.408 | 2.103 |
| Months since first SARS-CoV-2 infection | -0.000 | 0.001 | -0.446 | 0.656 | -0.002 | 0.002 |
| Constant | 55.790 | 1.204 | 46.324 | 0.000 | 53.416 | 58.165 |
|  | **Coef.** | **SE** | **t** | **p** | **95% CI** | |
| Persistent complaints ^a^ (reference = no) | -0.255 | 1.710 | -0.149 | 0.882 | -3.629 | 3.118 |
| Vaccinated at time of infection (reference = no) | 2.402 | 2.145 | 1.120 | 0.264 | -1.828 | 6.633 |
| Constant | 54.734 | 1.368 | 40.018 | 0.000 | 52.037 | 57.432 |
|  | **Coef.** | **SE** | **t** | **p** | **95% CI** | |
| Persistent complaints ^a^ (reference = no) | 0.176 | 1.822 | 0.097 | 0.923 | -3.416 | 3.768 |
| Clinically relevant symptoms of depression (HADS ≥8) | -3.543 | 2.207 | -1.605 | 0.110 | -7.895 | 0.808 |
| Constant | 55.908 | 1.193 | 46.861 | 0.000 | 53.556 | 58.260 |

## eTable 5i Controlled Oral Word Association Test

|  | **Coef.** | **SE** | **t** | **p** | **95% CI** | |
| --- | --- | --- | --- | --- | --- | --- |
| Persistent complaints ^a^ (reference = no) | -1.535 | 1.787 | -0.859 | 0.391 | -5.059 | 1.989 |
| Non-Dutch origin ^b^ (reference = Dutch origin) | -2.118 | 3.032 | -0.699 | 0.486 | -8.098 | 3.861 |
| Constant | 53.933 | 1.279 | 42.175 | 0.000 | 51.411 | 56.455 |
|  | **Coef.** | **SE** | **t** | **p** | **95% CI** | |
| Persistent complaints ^a^ (reference = no) | -1.438 | 1.726 | -0.833 | 0.406 | -4.842 | 1.965 |
| Number of comorbidities ^c^ (reference= no) | 1.188 | 1.010 | 1.176 | 0.241 | -0.803 | 3.178 |
| Constant | 53.056 | 1.420 | 37.370 | 0.000 | 50.257 | 55.855 |
|  | **Coef.** | **SE** | **t** | **p** | **95% CI** | |
| Persistent complaints ^a^ (reference = no) | -1.628 | 1.731 | -0.941 | 0.348 | -5.041 | 1.785 |
| Psychiatric comorbidity (reference= no) | 0.096 | 4.001 | 0.024 | 0.981 | -7.794 | 7.986 |
| Constant | 53.818 | 1.276 | 42.164 | 0.000 | 51.301 | 56.335 |
|  | **Coef.** | **SE** | **t** | **p** | **95% CI** | |
| Persistent complaints ^a^ (reference = no) | -1.581 | 1.738 | -0.910 | 0.364 | -5.010 | 1.848 |
| ApoE-ɛ4 genotype (reference = non ɛ4-carriers) | 1.264 | 1.849 | 0.683 | 0.495 | -2.384 | 4.912 |
| Constant | 53.203 | 1.436 | 37.050 | 0.000 | 50.371 | 56.036 |
|  | **Coef.** | **SE** | **t** | **p** | **95% CI** | |
| Persistent complaints ^a^ (reference = no) | -1.352 | 1.717 | -0.787 | 0.432 | -4.739 | 2.034 |
| Hospital admission for COVID-19 (reference = no) | 0.543 | 1.873 | 0.290 | 0.772 | -3.150 | 4.236 |
| Constant | 53.574 | 1.382 | 38.776 | 0.000 | 50.850 | 56.298 |
|  | **Coef.** | **SE** | **t** | **p** | **95% CI** | |
| Persistent complaints ^a^ (reference = no) | -1.465 | 1.729 | -0.848 | 0.398 | -4.874 | 1.943 |
| Months since first SARS-CoV-2 infection | 0.000 | 0.001 | 0.459 | 0.647 | -0.002 | 0.003 |
| Constant | 53.790 | 1.261 | 42.659 | 0.000 | 51.304 | 56.277 |
|  | **Coef.** | **SE** | **t** | **p** | **95% CI** | |
| Persistent complaints ^a^ (reference = no) | -0.970 | 1.833 | -0.529 | 0.597 | -4.586 | 2.645 |
| Vaccinated at time of infection (reference = no) | 1.823 | 2.299 | 0.793 | 0.429 | -2.712 | 6.357 |
| Constant | 53.039 | 1.466 | 36.179 | 0.000 | 50.148 | 55.931 |
|  | **Coef.** | **SE** | **t** | **p** | **95% CI** | |
| Persistent complaints ^a^ (reference = no) | -0.149 | 1.908 | -0.078 | 0.938 | -3.910 | 3.612 |
| Clinically relevant symptoms of depression (HADS ≥8) | -3.313 | 2.311 | -1.433 | 0.153 | -7.869 | 1.244 |
| Constant | 53.811 | 1.249 | 43.074 | 0.000 | 51.348 | 56.274 |

## eTable 5j Digit-span backward

|  | **Coef.** | **SE** | **t** | **p** | **95% CI** | |
| --- | --- | --- | --- | --- | --- | --- |
| Persistent complaints ^a^ (reference = no) | 1.097 | 1.704 | 0.644 | 0.520 | -2.264 | 4.459 |
| Non-Dutch origin ^b^ (reference = Dutch origin) | -2.286 | 2.892 | -0.790 | 0.430 | -7.989 | 3.418 |
| Constant | 53.109 | 1.220 | 43.540 | 0.000 | 50.703 | 55.514 |
|  | **Coef.** | **SE** | **t** | **p** | **95% CI** | |
| Persistent complaints ^a^ (reference = no) | 1.322 | 1.680 | 0.787 | 0.432 | -1.991 | 4.635 |
| Number of comorbidities ^c^ (reference= no) | -0.575 | 0.983 | -0.585 | 0.559 | -2.513 | 1.362 |
| Constant | 53.361 | 1.382 | 38.612 | 0.000 | 50.636 | 56.086 |
|  | **Coef.** | **SE** | **t** | **p** | **95% CI** | |
| Persistent complaints ^a^ (reference = no) | 1.129 | 1.676 | 0.673 | 0.502 | -2.177 | 4.434 |
| Psychiatric comorbidity (reference= no) | -0.373 | 3.875 | -0.096 | 0.923 | -8.015 | 7.268 |
| Constant | 53.009 | 1.236 | 42.881 | 0.000 | 50.571 | 55.447 |
|  | **Coef.** | **SE** | **t** | **p** | **95% CI** | |
| Persistent complaints ^a^ (reference = no) | 1.044 | 1.764 | 0.592 | 0.555 | -2.435 | 4.523 |
| ApoE-ɛ4 genotype (reference = non ɛ4-carriers) | 0.526 | 1.876 | 0.280 | 0.780 | -3.175 | 4.227 |
| Constant | 52.929 | 1.457 | 36.327 | 0.000 | 50.055 | 55.803 |
|  | **Coef.** | **SE** | **t** | **p** | **95% CI** | |
| Persistent complaints ^a^ (reference = no) | 1.454 | 1.684 | 0.863 | 0.389 | -1.866 | 4.774 |
| Hospital admission for COVID-19 (reference = no) | -0.811 | 1.837 | -0.441 | 0.659 | -4.432 | 2.811 |
| Constant | 53.302 | 1.355 | 39.343 | 0.000 | 50.631 | 55.973 |
|  | **Coef.** | **SE** | **t** | **p** | **95% CI** | |
| Persistent complaints ^a^ (reference = no) | 1.497 | 1.694 | 0.884 | 0.378 | -1.843 | 4.836 |
| Months since first SARS-CoV-2 infection | -0.001 | 0.001 | -0.666 | 0.506 | -0.003 | 0.001 |
| Constant | 52.983 | 1.236 | 42.879 | 0.000 | 50.547 | 55.419 |
|  | **Coef.** | **SE** | **t** | **p** | **95% CI** | |
| Persistent complaints ^a^ (reference = no) | 1.484 | 1.788 | 0.830 | 0.407 | -2.042 | 5.010 |
| Vaccinated at time of infection (reference = no) | 0.592 | 2.242 | 0.264 | 0.792 | -3.830 | 5.014 |
| Constant | 53.170 | 1.430 | 37.193 | 0.000 | 50.350 | 55.989 |
|  | **Coef.** | **SE** | **t** | **p** | **95% CI** | |
| Persistent complaints ^a^ (reference = no) | 1.543 | 1.880 | 0.821 | 0.413 | -2.164 | 5.250 |
| Clinically relevant symptoms of depression (HADS ≥8) | -0.197 | 2.278 | -0.086 | 0.931 | -4.688 | 4.295 |
| Constant | 53.056 | 1.231 | 43.085 | 0.000 | 50.628 | 55.483 |

## eTable 5k Stroop Color-word

|  | **Coef.** | **SE** | **t** | **p** | **95% CI** | |
| --- | --- | --- | --- | --- | --- | --- |
| Persistent complaints ^a^ (reference = no) | -2.132 | 1.529 | -1.394 | 0.165 | -5.146 | 0.883 |
| Non-Dutch origin ^b^ (reference = Dutch origin) | -2.527 | 2.594 | -0.974 | 0.331 | -7.643 | 2.589 |
| Constant | 50.361 | 1.094 | 46.033 | 0.000 | 48.203 | 52.518 |
|  | **Coef.** | **SE** | **t** | **p** | **95% CI** | |
| Persistent complaints ^a^ (reference = no) | -2.383 | 1.468 | -1.623 | 0.106 | -5.277 | 0.511 |
| Number of comorbidities ^c^ (reference= no) | 0.487 | 0.858 | 0.568 | 0.571 | -1.205 | 2.180 |
| Constant | 49.914 | 1.207 | 41.346 | 0.000 | 47.534 | 52.295 |
|  | **Coef.** | **SE** | **t** | **p** | **95% CI** | |
| Persistent complaints ^a^ (reference = no) | -2.429 | 1.480 | -1.641 | 0.102 | -5.347 | 0.490 |
| Psychiatric comorbidity (reference= no) | 0.723 | 3.422 | 0.211 | 0.833 | -6.024 | 7.470 |
| Constant | 50.192 | 1.091 | 45.984 | 0.000 | 48.039 | 52.344 |
|  | **Coef.** | **SE** | **t** | **p** | **95% CI** | |
| Persistent complaints ^a^ (reference = no) | -2.822 | 1.523 | -1.853 | 0.065 | -5.826 | 0.183 |
| ApoE-ɛ4 genotype (reference = non ɛ4-carriers) | 1.949 | 1.620 | 1.203 | 0.231 | -1.248 | 5.145 |
| Constant | 49.808 | 1.258 | 39.585 | 0.000 | 47.326 | 52.290 |
|  | **Coef.** | **SE** | **t** | **p** | **95% CI** | |
| Persistent complaints ^a^ (reference = no) | -2.329 | 1.454 | -1.602 | 0.111 | -5.195 | 0.537 |
| Hospital admission for COVID-19 (reference = no) | -1.449 | 1.585 | -0.914 | 0.362 | -4.575 | 1.677 |
| Constant | 50.603 | 1.169 | 43.275 | 0.000 | 48.297 | 52.908 |
|  | **Coef.** | **SE** | **t** | **p** | **95% CI** | |
| Persistent complaints ^a^ (reference = no) | -2.315 | 1.466 | -1.579 | 0.116 | -5.206 | 0.575 |
| Months since first SARS-CoV-2 infection | 0.000 | 0.001 | 0.388 | 0.699 | -0.001 | 0.002 |
| Constant | 50.189 | 1.069 | 46.930 | 0.000 | 48.081 | 52.298 |
|  | **Coef.** | **SE** | **t** | **p** | **95% CI** | |
| Persistent complaints ^a^ (reference = no) | -1.753 | 1.534 | -1.143 | 0.254 | -4.779 | 1.272 |
| Vaccinated at time of infection (reference = no) | 2.166 | 1.924 | 1.126 | 0.262 | -1.629 | 5.960 |
| Constant | 49.573 | 1.227 | 40.408 | 0.000 | 47.153 | 51.992 |
|  | **Coef.** | **SE** | **t** | **p** | **95% CI** | |
| Persistent complaints ^a^ (reference = no) | -1.131 | 1.615 | -0.700 | 0.485 | -4.315 | 2.053 |
| Clinically relevant symptoms of depression (HADS ≥8) | -3.184 | 1.957 | -1.627 | 0.105 | -7.042 | 0.674 |
| Constant | 50.220 | 1.058 | 47.481 | 0.000 | 48.135 | 52.306 |

## eTable 5l Trail Making Test B

|  | **Coef.** | **SE** | **t** | **p** | **95% CI** | |
| --- | --- | --- | --- | --- | --- | --- |
| Persistent complaints ^a^ (reference = no) | -3.296 | 1.507 | -2.187 | 0.030 | -6.268 | -0.324 |
| **Non-Dutch origin ^b^ (reference = Dutch origin)** | **-8.170** | **2.550** | **-3.203** | **0.002** | **-13.200** | **-3.140** |
| Constant | 55.388 | 1.081 | 51.219 | 0.000 | 53.255 | 57.521 |
|  | **Coef.** | **SE** | **t** | **p** | **95% CI** | |
| Persistent complaints ^a^ (reference = no) | -3.793 | 1.497 | -2.534 | 0.012 | -6.744 | -0.842 |
| Number of comorbidities ^c^ (reference= no) | -0.025 | 0.878 | -0.029 | 0.977 | -1.757 | 1.706 |
| Constant | 54.974 | 1.228 | 44.757 | 0.000 | 52.552 | 57.396 |
|  | **Coef.** | **SE** | **t** | **p** | **95% CI** | |
| Persistent complaints ^a^ (reference = no) | -3.904 | 1.504 | -2.595 | 0.010 | -6.870 | -0.938 |
| Psychiatric comorbidity (reference= no) | 2.738 | 3.468 | 0.789 | 0.431 | -4.101 | 9.577 |
| Constant | 54.814 | 1.112 | 49.290 | 0.000 | 52.621 | 57.007 |
|  | **Coef.** | **SE** | **t** | **p** | **95% CI** | |
| Persistent complaints ^a^ (reference = no) | -4.240 | 1.566 | -2.708 | 0.007 | -7.328 | -1.151 |
| ApoE-ɛ4 genotype (reference = non ɛ4-carriers) | 1.305 | 1.670 | 0.782 | 0.435 | -1.988 | 4.599 |
| Constant | 54.665 | 1.292 | 42.318 | 0.000 | 52.117 | 57.214 |
|  | **Coef.** | **SE** | **t** | **p** | **95% CI** | |
| Persistent complaints ^a^ (reference = no) | -3.955 | 1.469 | -2.692 | 0.008 | -6.851 | -1.058 |
| **Hospital admission for COVID-19 (reference = no)** | **-3.355** | **1.607** | **-2.088** | **0.038** | **-6.523** | **-0.186** |
| Constant | 56.003 | 1.180 | 47.458 | 0.000 | 53.676 | 58.330 |
|  | **Coef.** | **SE** | **t** | **p** | **95% CI** | |
| Persistent complaints ^a^ (reference = no) | -3.920 | 1.494 | -2.623 | 0.009 | -6.866 | -0.973 |
| Months since first SARS-CoV-2 infection | -0.000 | 0.001 | -0.296 | 0.767 | -0.002 | 0.002 |
| Constant | 54.962 | 1.093 | 50.298 | 0.000 | 52.808 | 57.117 |
|  | **Coef.** | **SE** | **t** | **p** | **95% CI** | |
| Persistent complaints ^a^ (reference = no) | -3.495 | 1.578 | -2.215 | 0.028 | -6.607 | -0.384 |
| Vaccinated at time of infection (reference = no) | 2.334 | 1.974 | 1.182 | 0.239 | -1.560 | 6.227 |
| Constant | 54.190 | 1.266 | 42.816 | 0.000 | 51.694 | 56.687 |
|  | **Coef.** | **SE** | **t** | **p** | **95% CI** | |
| Persistent complaints ^a^ (reference = no) | -2.348 | 1.637 | -1.434 | 0.153 | -5.577 | 0.880 |
| **Clinically relevant symptoms of depression (HADS ≥8)** | **-4.257** | **1.980** | **-2.150** | **0.033** | **-8.160** | **-0.353** |
| Constant | 55.078 | 1.076 | 51.202 | 0.000 | 52.957 | 57.199 |

## eTable 5m Trail Making Test B|A

|  | **Coef.** | **SE** | **t** | **p** | **95% CI** | |
| --- | --- | --- | --- | --- | --- | --- |
| Persistent complaints ^a^ (reference = no) | -3.686 | 1.385 | -2.662 | 0.008 | -6.417 | -0.955 |
| Non-Dutch origin ^b^ (reference = Dutch origin) | -3.456 | 2.344 | -1.474 | 0.142 | -8.079 | 1.167 |
| Constant | 52.435 | 0.994 | 52.759 | 0.000 | 50.474 | 54.395 |
|  | **Coef.** | **SE** | **t** | **p** | **95% CI** | |
| Persistent complaints ^a^ (reference = no) | -3.885 | 1.337 | -2.906 | 0.004 | -6.521 | -1.249 |
| Number of comorbidities ^c^ (reference= no) | -0.802 | 0.784 | -1.023 | 0.308 | -2.349 | 0.744 |
| Constant | 52.759 | 1.097 | 48.089 | 0.000 | 50.596 | 54.923 |
|  | **Coef.** | **SE** | **t** | **p** | **95% CI** | |
| Persistent complaints ^a^ (reference = no) | -3.996 | 1.343 | -2.976 | 0.003 | -6.644 | -1.348 |
| Psychiatric comorbidity (reference= no) | 3.953 | 3.096 | 1.277 | 0.203 | -2.151 | 10.058 |
| Constant | 52.045 | 0.993 | 52.425 | 0.000 | 50.087 | 54.002 |
|  | **Coef.** | **SE** | **t** | **p** | **95% CI** | |
| Persistent complaints ^a^ (reference = no) | -4.258 | 1.398 | -3.047 | 0.003 | -7.014 | -1.501 |
| ApoE-ɛ4 genotype (reference = non ɛ4-carriers) | 0.712 | 1.490 | 0.478 | 0.633 | -2.228 | 3.653 |
| Constant | 52.283 | 1.153 | 45.345 | 0.000 | 50.009 | 54.558 |
|  | **Coef.** | **SE** | **t** | **p** | **95% CI** | |
| Persistent complaints ^a^ (reference = no) | -4.065 | 1.320 | -3.079 | 0.002 | -6.668 | -1.461 |
| Hospital admission for COVID-19 (reference = no) | -2.381 | 1.444 | -1.649 | 0.101 | -5.228 | 0.467 |
| Constant | 53.021 | 1.061 | 49.991 | 0.000 | 50.930 | 55.112 |
|  | **Coef.** | **SE** | **t** | **p** | **95% CI** | |
| Persistent complaints ^a^ (reference = no) | -4.042 | 1.339 | -3.019 | 0.003 | -6.682 | -1.402 |
| Months since first SARS-CoV-2 infection | 0.000 | 0.001 | 0.125 | 0.901 | -0.001 | 0.002 |
| Constant | 52.312 | 0.979 | 53.438 | 0.000 | 50.382 | 54.243 |
|  | **Coef.** | **SE** | **t** | **p** | **95% CI** | |
| Persistent complaints ^a^ (reference = no) | -4.019 | 1.407 | -2.857 | 0.005 | -6.793 | -1.245 |
| Vaccinated at time of infection (reference = no) | 1.270 | 1.760 | 0.722 | 0.471 | -2.201 | 4.741 |
| Constant | 51.967 | 1.128 | 46.057 | 0.000 | 49.741 | 54.192 |
|  | **Coef.** | **SE** | **t** | **p** | **95% CI** | |
| Persistent complaints ^a^ (reference = no) | -2.967 | 1.473 | -2.014 | 0.045 | -5.871 | -0.063 |
| Clinically relevant symptoms of depression (HADS ≥8) | -2.905 | 1.781 | -1.632 | 0.104 | -6.416 | 0.606 |
| Constant | 52.363 | 0.968 | 54.118 | 0.000 | 50.455 | 54.270 |

## eTable 5n Rey Auditory Verbal Learning test immediate

|  | **Coef.** | **SE** | **t** | **p** | **95% CI** | |
| --- | --- | --- | --- | --- | --- | --- |
| Persistent complaints ^a^ (reference = no) | 1.062 | 1.666 | 0.638 | 0.524 | -2.223 | 4.347 |
| Non-Dutch origin ^b^ (reference = Dutch origin) | -3.709 | 2.826 | -1.313 | 0.191 | -9.283 | 1.864 |
| Constant | 45.506 | 1.192 | 38.176 | 0.000 | 43.155 | 47.857 |
|  | **Coef.** | **SE** | **t** | **p** | **95% CI** | |
| Persistent complaints ^a^ (reference = no) | 0.631 | 1.613 | 0.391 | 0.696 | -2.549 | 3.811 |
| Number of comorbidities ^c^ (reference= no) | -1.207 | 0.943 | -1.280 | 0.202 | -3.067 | 0.652 |
| Constant | 46.092 | 1.326 | 34.752 | 0.000 | 43.477 | 48.707 |
|  | **Coef.** | **SE** | **t** | **p** | **95% CI** | |
| Persistent complaints ^a^ (reference = no) | 0.441 | 1.623 | 0.272 | 0.786 | -2.759 | 3.641 |
| Psychiatric comorbidity (reference= no) | -4.254 | 3.752 | -1.134 | 0.258 | -11.652 | 3.143 |
| Constant | 45.534 | 1.197 | 38.050 | 0.000 | 43.174 | 47.894 |
|  | **Coef.** | **SE** | **t** | **p** | **95% CI** | |
| Persistent complaints ^a^ (reference = no) | 0.907 | 1.661 | 0.546 | 0.586 | -2.369 | 4.182 |
| ApoE-ɛ4 genotype (reference = non ɛ4-carriers) | 1.724 | 1.767 | 0.976 | 0.330 | -1.761 | 5.209 |
| Constant | 44.368 | 1.372 | 32.342 | 0.000 | 41.662 | 47.074 |
|  | **Coef.** | **SE** | **t** | **p** | **95% CI** | |
| Persistent complaints ^a^ (reference = no) | 0.557 | 1.606 | 0.347 | 0.729 | -2.610 | 3.723 |
| Hospital admission for COVID-19 (reference = no) | 0.114 | 1.751 | 0.065 | 0.948 | -3.339 | 3.568 |
| Constant | 45.356 | 1.292 | 35.108 | 0.000 | 42.809 | 47.904 |
|  | **Coef.** | **SE** | **t** | **p** | **95% CI** | |
| Persistent complaints ^a^ (reference = no) | 0.549 | 1.607 | 0.341 | 0.733 | -2.621 | 3.718 |
| Months since first SARS-CoV-2 infection | -0.001 | 0.001 | -1.014 | 0.312 | -0.003 | 0.001 |
| Constant | 45.293 | 1.173 | 38.627 | 0.000 | 42.981 | 47.605 |
|  | **Coef.** | **SE** | **t** | **p** | **95% CI** | |
| Persistent complaints ^a^ (reference = no) | 0.603 | 1.692 | 0.356 | 0.722 | -2.734 | 3.940 |
| Vaccinated at time of infection (reference = no) | 0.575 | 2.122 | 0.271 | 0.787 | -3.610 | 4.760 |
| Constant | 45.327 | 1.353 | 33.499 | 0.000 | 42.658 | 47.995 |
|  | **Coef.** | **SE** | **t** | **p** | **95% CI** | |
| Persistent complaints ^a^ (reference = no) | 1.377 | 1.788 | 0.770 | 0.442 | -2.148 | 4.901 |
| Clinically relevant symptoms of depression (HADS ≥8) | -2.242 | 2.166 | -1.035 | 0.302 | -6.512 | 2.028 |
| Constant | 45.438 | 1.171 | 38.813 | 0.000 | 43.130 | 47.746 |

## eTable 5o Rey Auditory Verbal Learning test recall

|  | **Coef.** | **SE** | **t** | **p** | **95% CI** | |
| --- | --- | --- | --- | --- | --- | --- |
| Persistent complaints ^a^ (reference = no) | 1.847 | 1.649 | 1.120 | 0.264 | -1.406 | 5.099 |
| Non-Dutch origin ^b^ (reference = Dutch origin) | -2.574 | 2.798 | -0.920 | 0.359 | -8.092 | 2.945 |
| Constant | 45.634 | 1.180 | 38.664 | 0.000 | 43.306 | 47.962 |
|  | **Coef.** | **SE** | **t** | **p** | **95% CI** | |
| Persistent complaints ^a^ (reference = no) | 1.738 | 1.601 | 1.086 | 0.279 | -1.418 | 4.895 |
| Number of comorbidities ^c^ (reference= no) | -0.307 | 0.936 | -0.328 | 0.743 | -2.153 | 1.539 |
| Constant | 45.698 | 1.317 | 34.709 | 0.000 | 43.103 | 48.294 |
|  | **Coef.** | **SE** | **t** | **p** | **95% CI** | |
| Persistent complaints ^a^ (reference = no) | 1.492 | 1.604 | 0.930 | 0.353 | -1.671 | 4.655 |
| Psychiatric comorbidity (reference= no) | 1.850 | 3.708 | 0.499 | 0.618 | -5.462 | 9.162 |
| Constant | 45.404 | 1.183 | 38.384 | 0.000 | 43.071 | 47.736 |
|  | **Coef.** | **SE** | **t** | **p** | **95% CI** | |
| Persistent complaints ^a^ (reference = no) | 2.395 | 1.655 | 1.447 | 0.150 | -0.871 | 5.660 |
| ApoE-ɛ4 genotype (reference = non ɛ4-carriers) | 0.851 | 1.761 | 0.483 | 0.629 | -2.623 | 4.325 |
| Constant | 44.532 | 1.367 | 32.565 | 0.000 | 41.835 | 47.230 |
|  | **Coef.** | **SE** | **t** | **p** | **95% CI** | |
| Persistent complaints ^a^ (reference = no) | 1.762 | 1.584 | 1.112 | 0.267 | -1.361 | 4.885 |
| Hospital admission for COVID-19 (reference = no) | 2.004 | 1.728 | 1.160 | 0.247 | -1.402 | 5.410 |
| Constant | 44.958 | 1.274 | 35.281 | 0.000 | 42.445 | 47.470 |
|  | **Coef.** | **SE** | **t** | **p** | **95% CI** | |
| Persistent complaints ^a^ (reference = no) | 1.592 | 0.988 | 0.324 | -1.566 | 4.712 |  |
| Months since first SARS-CoV-2 infection | -0.000 | 0.001 | -0.040 | 0.968 | -0.002 | 0.002 |
| Constant | 45.573 | 1.161 | 39.241 | 0.000 | 43.284 | 47.863 |
|  | **Coef.** | **SE** | **t** | **p** | **95% CI** | |
| Persistent complaints ^a^ (reference = no) | 1.587 | 1.677 | 0.946 | 0.345 | -1.720 | 4.894 |
| Vaccinated at time of infection (reference = no) | 1.167 | 2.103 | 0.555 | 0.580 | -2.981 | 5.314 |
| Constant | 45.638 | 1.341 | 34.033 | 0.000 | 42.993 | 48.282 |
|  | **Coef.** | **SE** | **t** | **p** | **95% CI** | |
| Persistent complaints ^a^ (reference = no) | 2.406 | 1.770 | 1.359 | 0.176 | -1.084 | 5.896 |
| Clinically relevant symptoms of depression (HADS ≥8) | -1.871 | 2.145 | -0.872 | 0.384 | -6.099 | 2.358 |
| Constant | 45.616 | 1.159 | 39.346 | 0.000 | 43.330 | 47.902 |

## eTable 5p Rey Complex Figure Test recall condition

|  | **Coef.** | **SE** | **t** | **p** | **95% CI** | |
| --- | --- | --- | --- | --- | --- | --- |
| Persistent complaints ^a^ (reference = no) | -0.793 | 0.869 | -0.912 | 0.363 | -2.506 | 0.921 |
| Age in years | -0.128 | 0.032 | -3.968 | 0.000 | -0.192 | -0.065 |
| Sex (reference = male) | -1.946 | 0.905 | -2.150 | 0.033 | -3.731 | -0.160 |
| Highest level of education (reference = low) | 2.529 | 0.732 | 3.455 | 0.001 | 1.085 | 3.973 |
| Non-Dutch origin ^b^ (reference = Dutch origin) | 0.948 | 1.387 | 0.683 | 0.495 | -1.789 | 3.685 |
| Constant | 26.689 | 3.743 | 7.131 | 0.000 | 19.307 | 34.071 |
|  | **Coef.** | **SE** | **t** | **p** | **95% CI** | |
| Persistent complaints ^a^ (reference = no) | -0.659 | 0.846 | -0.778 | 0.437 | -2.328 | 1.011 |
| Age in years | -0.125 | 0.033 | -3.759 | 0.000 | -0.190 | -0.059 |
| Sex (reference = male) | -1.865 | 0.887 | -2.103 | 0.037 | -3.614 | -0.116 |
| Highest level of education (reference = low) | 2.416 | 0.725 | 3.331 | 0.001 | 0.986 | 3.847 |
| Number of comorbidities ^c^ (reference= no) | 0.058 | 0.492 | 0.118 | 0.906 | -0.913 | 1.029 |
| Constant | 26.674 | 3.669 | 7.269 | 0.000 | 19.438 | 33.910 |
|  | **Coef.** | **SE** | **t** | **p** | **95% CI** | |
| Persistent complaints ^a^ (reference = no) | -0.657 | 0.848 | -0.774 | 0.440 | -2.330 | 1.016 |
| Age in years | -0.126 | 0.032 | -3.933 | 0.000 | -0.189 | -0.063 |
| Sex (reference = male) | -2.052 | 0.910 | -2.254 | 0.025 | -3.848 | -0.257 |
| Highest level of education (reference = low) | 2.383 | 0.718 | 3.320 | 0.001 | 0.967 | 3.798 |
| Psychiatric comorbidity (reference= no) | 1.398 | 1.868 | 0.748 | 0.455 | -2.287 | 5.082 |
| Constant | 27.057 | 3.695 | 7.323 | 0.000 | 19.771 | 34.343 |
|  | **Coef.** | **SE** | **t** | **p** | **95% CI** | |
| Persistent complaints ^a^ (reference = no) | 0.840 | -0.726 | 0.469 | -2.266 | 1.046 |  |
| Age in years | -0.116 | 0.031 | -3.701 | 0.000 | -0.178 | -0.054 |
| Sex (reference = male) | -1.853 | 0.870 | -2.130 | 0.034 | -3.569 | -0.138 |
| Highest level of education (reference = low) | 2.452 | 0.710 | 3.454 | 0.001 | 1.052 | 3.852 |
| ApoE-ɛ4 genotype (reference = non ɛ4-carriers) | 1.517 | 0.843 | 1.798 | 0.074 | -0.147 | 3.180 |
| Constant | 25.664 | 3.639 | 7.052 | 0.000 | 18.488 | 32.840 |
|  | **Coef.** | **SE** | **t** | **p** | **95% CI** | |
| Persistent complaints ^a^ (reference = no) | -0.595 | 0.840 | -0.708 | 0.479 | -2.251 | 1.061 |
| Age in years | -0.102 | 0.033 | -3.141 | 0.002 | -0.166 | -0.038 |
| Sex (reference = male) | -1.961 | 0.873 | -2.246 | 0.026 | -3.683 | -0.239 |
| Highest level of education (reference = low) | 2.258 | 0.717 | 3.152 | 0.002 | 0.845 | 3.671 |
| Hospital admission for COVID-19 (reference = no) | -1.701 | 0.911 | -1.868 | 0.063 | -3.497 | 0.095 |
| Constant | 26.553 | 3.626 | 7.324 | 0.000 | 19.404 | 33.702 |
|  | **Coef.** | **SE** | **t** | **p** | **95% CI** | |
| Persistent complaints ^a^ (reference = no) | -0.681 | 0.848 | -0.803 | 0.423 | -2.354 | 0.991 |
| Age in years | -0.116 | 0.032 | -3.671 | 0.000 | -0.179 | -0.054 |
| Sex (reference = male) | -1.875 | 0.878 | -2.135 | 0.034 | -3.607 | -0.144 |
| Highest level of education (reference = low) | 2.413 | 0.716 | 3.372 | 0.001 | 1.002 | 3.824 |
| Months since first SARS-CoV-2 infection | -0.000 | 0.000 | -1.011 | 0.313 | -0.001 | 0.000 |
| Constant | 26.273 | 3.654 | 7.190 | 0.000 | 19.068 | 33.478 |
|  | **Coef.** | **SE** | **t** | **p** | **95% CI** | |
| Persistent complaints ^a^ (reference = no) | -0.699 | 0.871 | -0.802 | 0.424 | -2.418 | 1.020 |
| Age in years | -0.125 | 0.033 | -3.816 | 0.000 | -0.190 | -0.061 |
| Sex (reference = male) | -1.922 | 0.900 | -2.136 | 0.034 | -3.696 | -0.147 |
| Highest level of education (reference = low) | 2.208 | 0.755 | 2.925 | 0.004 | 0.719 | 3.698 |
| **Vaccinated at time of infection (reference = no)** | **2.240** | **1.045** | **2.143** | **0.033** | **0.179** | **4.301** |
| Constant | 26.997 | 3.794 | 7.116 | 0.000 | 19.514 | 34.480 |
|  | **Coef.** | **SE** | **t** | **p** | **95% CI** | |
| Persistent complaints ^a^ (reference = no) | -0.739 | 0.910 | -0.812 | 0.418 | -2.534 | 1.056 |
| Age in years | -0.119 | 0.032 | -3.748 | 0.000 | -0.181 | -0.056 |
| Sex (reference = male) | -1.794 | 0.877 | -2.045 | 0.042 | -3.524 | -0.065 |
| Highest level of education (reference = low) | 2.449 | 0.716 | 3.418 | 0.001 | 1.036 | 3.861 |
| Clinically relevant symptoms of depression (HADS ≥8) | -0.049 | 1.042 | -0.047 | 0.963 | -2.104 | 2.006 |
| Constant | 26.254 | 3.653 | 7.186 | 0.000 | 19.050 | 33.457 |

## eTable 5q Animal Fluency

|  | **Coef.** | **SE** | **t** | **p** | **95% CI** | |
| --- | --- | --- | --- | --- | --- | --- |
| Persistent complaints ^a^ (reference = no) | 0.949 | 1.557 | 0.610 | 0.543 | -2.122 | 4.021 |
| **Non-Dutch origin ^b^ (reference = Dutch origin)** | **-6.857** | **2.642** | **-2.595** | **0.010** | **-12.068** | **-1.646** |
| Constant | 55.315 | 1.115 | 49.631 | 0.000 | 53.117 | 57.514 |
|  | **Coef.** | **SE** | **t** | **p** | **95% CI** | |
| Persistent complaints ^a^ (reference = no) | 0.669 | 1.533 | 0.436 | 0.663 | -2.354 | 3.692 |
| Number of comorbidities ^c^ (reference= no) | -1.269 | 0.897 | -1.415 | 0.159 | -3.036 | 0.499 |
| Constant | 55.778 | 1.261 | 44.240 | 0.000 | 53.292 | 58.264 |
|  | **Coef.** | **SE** | **t** | **p** | **95% CI** | |
| Persistent complaints ^a^ (reference = no) | 0.281 | 1.523 | 0.184 | 0.854 | -2.721 | 3.283 |
| Psychiatric comorbidity (reference= no) | -5.062 | 3.520 | -1.438 | 0.152 | -12.003 | 1.878 |
| Constant | 55.222 | 1.123 | 49.181 | 0.000 | 53.008 | 57.436 |
|  | **Coef.** | **SE** | **t** | **p** | **95% CI** | |
| Persistent complaints ^a^ (reference = no) | 0.258 | 1.608 | 0.160 | 0.873 | -2.914 | 3.430 |
| ApoE-ɛ4 genotype (reference = non ɛ4-carriers) | 0.586 | 1.711 | 0.342 | 0.732 | -2.789 | 3.960 |
| Constant | 54.863 | 1.328 | 41.301 | 0.000 | 52.243 | 57.483 |
|  | **Coef.** | **SE** | **t** | **p** | **95% CI** | |
| Persistent complaints ^a^ (reference = no) | 0.472 | 1.526 | 0.309 | 0.758 | -2.536 | 3.480 |
| Hospital admission for COVID-19 (reference = no) | -1.755 | 1.664 | -1.055 | 0.293 | -5.036 | 1.525 |
| Constant | 55.512 | 1.227 | 45.231 | 0.000 | 53.092 | 57.932 |
|  | **Coef.** | **SE** | **t** | **p** | **95% CI** | |
| Persistent complaints ^a^ (reference = no) | 0.458 | 1.540 | 0.298 | 0.766 | -2.578 | 3.495 |
| Months since first SARS-CoV-2 infection | 0.000 | 0.001 | 0.078 | 0.938 | -0.002 | 0.002 |
| Constant | 54.976 | 1.123 | 48.938 | 0.000 | 52.761 | 57.191 |
|  | **Coef.** | **SE** | **t** | **p** | **95% CI** | |
| Persistent complaints ^a^ (reference = no) | 0.750 | 1.625 | 0.462 | 0.645 | -2.455 | 3.955 |
| Vaccinated at time of infection (reference = no) | 1.973 | 2.038 | 0.968 | 0.334 | -2.046 | 5.993 |
| Constant | 54.366 | 1.299 | 41.837 | 0.000 | 51.803 | 56.929 |
|  | **Coef.** | **SE** | **t** | **p** | **95% CI** | |
| Persistent complaints ^a^ (reference = no) | 1.785 | 1.695 | 1.053 | 0.294 | -1.558 | 5.127 |
| Clinically relevant symptoms of depression (HADS ≥8) | -3.479 | 2.054 | -1.694 | 0.092 | -7.529 | 0.570 |
| Constant | 55.041 | 1.110 | 49.573 | 0.000 | 52.852 | 57.230 |

## eTable 5r Rey Complex Figure Test copy condition

|  | **Coef.** | **SE** | **t** | **p** | **95% CI** | |
| --- | --- | --- | --- | --- | --- | --- |
| Persistent complaints ^a^ (reference = no) | -0.495 | 0.293 | -1.689 | 0.093 | -1.073 | 0.083 |
| Age in years | -0.038 | 0.011 | -3.529 | 0.001 | -0.060 | -0.017 |
| Sex (reference = male) | 0.122 | 0.305 | 0.399 | 0.690 | -0.481 | 0.724 |
| Highest level of education (reference = low) | 1.052 | 0.247 | 4.258 | 0.000 | 0.564 | 1.539 |
| Non-Dutch origin ^b^ (reference = Dutch origin) | 0.252 | 0.468 | 0.539 | 0.590 | -0.671 | 1.176 |
| Constant | 34.074 | 1.263 | 26.989 | 0.000 | 31.584 | 36.564 |
|  | **Coef.** | **SE** | **t** | **p** | **95% CI** | |
| Persistent complaints ^a^ (reference = no) | -0.480 | 0.297 | -1.615 | 0.108 | -1.067 | 0.106 |
| Age in years | -0.029 | 0.012 | -2.494 | 0.013 | -0.052 | -0.006 |
| Sex (reference = male) | 0.131 | 0.312 | 0.422 | 0.674 | -0.483 | 0.746 |
| Highest level of education (reference = low) | 0.927 | 0.255 | 3.636 | 0.000 | 0.424 | 1.430 |
| Number of comorbidities ^c^ (reference= no) | -0.177 | 0.173 | -1.023 | 0.307 | -0.518 | 0.164 |
| Constant | 33.965 | 1.290 | 26.339 | 0.000 | 31.422 | 36.507 |
|  | **Coef.** | **SE** | **t** | **p** | **95% CI** | |
| Persistent complaints ^a^ (reference = no) | -0.524 | 0.299 | -1.752 | 0.081 | -1.115 | 0.066 |
| Age in years | -0.032 | 0.011 | -2.833 | 0.005 | -0.054 | -0.010 |
| Sex (reference = male) | 0.139 | 0.321 | 0.432 | 0.666 | -0.495 | 0.772 |
| Highest level of education (reference = low) | 0.963 | 0.253 | 3.803 | 0.000 | 0.464 | 1.462 |
| Psychiatric comorbidity (reference= no) | -0.371 | 0.659 | -0.562 | 0.575 | -1.671 | 0.930 |
| Constant | 33.934 | 1.304 | 26.028 | 0.000 | 31.363 | 36.505 |
|  | **Coef.** | **SE** | **t** | **p** | **95% CI** | |
| Persistent complaints ^a^ (reference = no) | -0.501 | 0.296 | -1.690 | 0.093 | -1.085 | 0.083 |
| Age in years | -0.033 | 0.011 | -2.937 | 0.004 | -0.054 | -0.011 |
| Sex (reference = male) | 0.081 | 0.307 | 0.265 | 0.791 | -0.524 | 0.687 |
| Highest level of education (reference = low) | 0.978 | 0.250 | 3.905 | 0.000 | 0.484 | 1.472 |
| ApoE-ɛ4 genotype (reference = non ɛ4-carriers) | 0.098 | 0.298 | 0.328 | 0.743 | -0.489 | 0.684 |
| Constant | 33.967 | 1.284 | 26.455 | 0.000 | 31.435 | 36.499 |
|  | **Coef.** | **SE** | **t** | **p** | **95% CI** | |
| Persistent complaints ^a^ (reference = no) | -0.419 | 0.290 | -1.445 | 0.150 | -0.990 | 0.153 |
| Age in years | -0.023 | 0.011 | -2.080 | 0.039 | -0.045 | -0.001 |
| Sex (reference = male) | -0.009 | 0.301 | -0.031 | 0.975 | -0.604 | 0.585 |
| Highest level of education (reference = low) | 0.870 | 0.247 | 3.518 | 0.001 | 0.382 | 1.357 |
| **Hospital admission for COVID-19 (reference = no)** | **-0.971** | **0.314** | **-3.089** | **0.002** | **-1.591** | **-0.351** |
| Constant | 34.175 | 1.251 | 27.317 | 0.000 | 31.708 | 36.642 |
|  | **Coef.** | **SE** | **t** | **p** | **95% CI** | |
| Persistent complaints ^a^ (reference = no) | -0.514 | 0.298 | -1.726 | 0.086 | -1.101 | 0.073 |
| Age in years | -0.033 | 0.011 | -2.936 | 0.004 | -0.055 | -0.011 |
| Sex (reference = male) | 0.077 | 0.308 | 0.248 | 0.804 | -0.531 | 0.685 |
| Highest level of education (reference = low) | 0.972 | 0.251 | 3.868 | 0.000 | 0.476 | 1.467 |
| Months since first SARS-CoV-2 infection | -0.000 | 0.000 | -0.087 | 0.931 | -0.000 | 0.000 |
| Constant | 34.029 | 1.283 | 26.525 | 0.000 | 31.500 | 36.559 |
|  | **Coef.** | **SE** | **t** | **p** | **95% CI** | |
| Persistent complaints ^a^ (reference = no) | -0.462 | 0.303 | -1.526 | 0.129 | -1.059 | 0.135 |
| Age in years | -0.039 | 0.011 | -3.394 | 0.001 | -0.061 | -0.016 |
| Sex (reference = male) | 0.001 | 0.312 | 0.002 | 0.999 | -0.616 | 0.617 |
| Highest level of education (reference = low) | 0.917 | 0.262 | 3.498 | 0.001 | 0.400 | 1.435 |
| **Vaccinated at time of infection (reference = no)** | **0.778** | **0.363** | **2.144** | **0.033** | **0.062** | **1.494** |
| Constant | 34.411 | 1.318 | 26.114 | 0.000 | 31.812 | 37.010 |
|  | **Coef.** | **SE** | **t** | **p** | **95% CI** | |
| Persistent complaints ^a^ (reference = no) | -0.579 | 0.318 | -1.819 | 0.070 | -1.207 | 0.049 |
| Age in years | -0.032 | 0.011 | -2.921 | 0.004 | -0.054 | -0.011 |
| Sex (reference = male) | 0.077 | 0.307 | 0.252 | 0.801 | -0.528 | 0.682 |
| Highest level of education (reference = low) | 0.970 | 0.251 | 3.871 | 0.000 | 0.476 | 1.464 |
| Clinically relevant symptoms of depression (HADS ≥8) | 0.209 | 0.365 | 0.574 | 0.567 | -0.510 | 0.928 |
| Constant | 34.009 | 1.278 | 26.611 | 0.000 | 31.489 | 36.529 |

^a^ Complaints consist of persistent severe fatigue (CIS fatigue ≥35) and difficulty concentrating (CIS concentration ≥18). ^b^ Participants were considered of non-Dutch ethnic origin if they were born outside the Netherlands and at least one parent was born outside the Netherlands; or they were born in the Netherlands but both parents were born outside the Netherlands. ^c^ Comorbidities include cardiovascular disease, chronic pulmonary disease, diabetes, neurological disease, haematological disease, rheumatic disease, thyroid disorders, renal disease, liver disease, cancer, and psychiatric illness. Significant p-values are in bold.

Abbreviations: HADS, The Hospital Anxiety and Depression Scale.
